# DNA methylation changes measured in pre‐diagnostic peripheral blood samples are associated with smoking and lung cancer risk

**DOI:** 10.1002/ijc.30431

**Published:** 2016-10-11

**Authors:** Laura Baglietto, Erica Ponzi, Philip Haycock, Allison Hodge, Manuela Bianca Assumma, Chol‐Hee Jung, Jessica Chung, Francesca Fasanelli, Florence Guida, Gianluca Campanella, Marc Chadeau‐Hyam, Kjell Grankvist, Mikael Johansson, Ugo Ala, Paolo Provero, Ee Ming Wong, Jihoon Joo, Dallas R. English, Nabila Kazmi, Eiliv Lund, Christian Faltus, Rudolf Kaaks, Angela Risch, Myrto Barrdahl, Torkjel M. Sandanger, Melissa C. Southey, Graham G. Giles, Mattias Johansson, Paolo Vineis, Silvia Polidoro, Caroline L. Relton, Gianluca Severi

**Affiliations:** ^1^ Université Paris‐Saclay, Univ. Paris‐Sud, UVSQ, CESP, INSERM Villejuif France; ^2^ Gustave Roussy Villejuif F‐94805 France; ^3^ Cancer Epidemiology Centre Cancer Council Victoria Melbourne Australia; ^4^ Centre for Epidemiology and Biostatistics, Melbourne School of Population & Global Health The University of Melbourne Australia; ^5^ Department of Evolutionary Biology and Environmental Studies University of Zurich Switzerland; ^6^ Epidemiology, Biostatistics and Prevention Institute, University of Zurich Switzerland; ^7^ MRC Integrative Epidemiology Unit, School of Social & Community Medicine University of Bristol BS8 2BN UK; ^8^ HuGeF, Human Genetics Foundation Torino 10126 Italy; ^9^ Victorian Life Sciences Computation Initiative The University of Melbourne Victoria 3010 Australia; ^10^ Unit of Cancer Epidemiology, Citta' della Salute e della Scienza Hospital‐University of Turin and Center for Cancer Prevention (CPO) 10126 Torino; ^11^ MRC‐PHE Centre for Environment and Health, Department of Epidemiology and Biostatistics, School of Public Health Imperial College London Norfolk Place London W2 1PG UK; ^12^ Department of Biobank Research Umeå University Sweden; ^13^ Department of Radiation Sciences Umeå University Sweden; ^14^ Department of Molecular Biotechnology and Health Sciences Università di Torino 10126 Italy; ^15^ Center for Translational Genomics and Bioinformatics, San Raffaele Scientific Institute Milan Italy; ^16^ Genetic Epidemiology Laboratory, Department of Pathology The University of Melbourne Australia; ^17^ Department of Community Medicine UiT‐ The Arctic University of Norway Tromso Norway; ^18^ Division of Cancer Research and Epigenetics, Department of Molecular Biology University of Salzburg Salzburg Austria; ^19^ Division of Epigenomics and Cancer Risk Factors DKFZ – German Cancer Research Center Heidelberg Germany; ^20^ Division of Cancer Epidemiology DKFZ ‐ German Cancer Research Center Heidelberg Germany; ^21^ Translational Lung Research Center Heidelberg (TLRC‐H), Member of the German Center for Lung Research (DZL) Heidelberg Germany; ^22^ International Agency for Research on Cancer Lyon France

**Keywords:** DNA methylation, lung cancer risk, smoking

## Abstract

DNA methylation changes are associated with cigarette smoking. We used the Illumina Infinium HumanMethylation450 array to determine whether methylation in DNA from pre‐diagnostic, peripheral blood samples is associated with lung cancer risk. We used a case‐control study nested within the EPIC‐Italy cohort and a study within the MCCS cohort as discovery sets (a total of 552 case‐control pairs). We validated the top signals in 429 case‐control pairs from another 3 studies. We identified six CpGs for which hypomethylation was associated with lung cancer risk: cg05575921 in the *AHRR* gene (*p*‐value_pooled_ = 4 × 10^−17^), cg03636183 in the *F2RL3* gene (*p*‐value_pooled_ = 2 × 10 ^− 13^), cg21566642 and cg05951221 in 2q37.1 (*p*‐value_pooled_ = 7 × 10^−16^ and 1 × 10^−11^ respectively), cg06126421 in 6p21.33 (*p*‐value_pooled_ = 2 × 10^−15^) and cg23387569 in 12q14.1 (*p*‐value_pooled_ = 5 × 10^−7^). For cg05951221 and cg23387569 the strength of association was virtually identical in never and current smokers. For all these CpGs except for cg23387569, the methylation levels were different across smoking categories in controls (*p*‐values_heterogeneity_ ≤ 1.8 x10 ^− 7^), were lowest for current smokers and increased with time since quitting for former smokers. We observed a gain in discrimination between cases and controls measured by the area under the ROC curve of at least 8% (*p*‐values ≥ 0.003) in former smokers by adding methylation at the 6 CpGs into risk prediction models including smoking status and number of pack‐years. Our findings provide convincing evidence that smoking and possibly other factors lead to DNA methylation changes measurable in peripheral blood that may improve prediction of lung cancer risk.

## Abstract 2

**What's new?**

It is well known that smoking can cause lung cancer but the concept that it might do so by changing DNA methylation is only emerging. Here the authors identify six sites of methylation (CpGs), where methylation levels were associated with lung cancer risk after adjusting for smoking, current or former. Methylation of five of the CpGs was lowest in current smokers and increased in former smokers with time since quitting, supporting the growing evidence that smoking may lead to DNA methylation changes measurable in peripheral blood and useful as predictive markers for lung cancer risk, especially in former smokers.

Smoking is the main cause of lung cancer with attributable risks of at least 85% for men and 60% for women,^1^, ^2^ yet a significant number of cases cannot be attributed either to cigarette smoking or other established risk factors such as air pollution. The major mechanisms considered to explain the effect of cigarette smoking on lung cancer risk include the exposure to carcinogenic compounds, the formation of DNA adducts and the accumulation of permanent somatic mutations in tumor suppressor genes and dominant oncogenes^3^. The balance between metabolic activation and detoxification of carcinogens varies between individuals and this is likely to be at least partly responsible for the variation in susceptibility to lung cancer among smokers.

Alterations of the methylation profile of DNA from peripheral blood associated with cigarette smoking have been recently described^4^, ^5^, ^6^, ^7^, ^8^, ^9^, ^10^, ^11^. The altered DNA methylation levels persist long after smoking cessation for some genomic locations (i.e. CpGs) while for others return to those of never‐smokers^12^. Recently, using a study conducted within the NOWAC cohort as discovery data set and studies within the Australian MCCS cohort, the Swedish NSHDS and the EPIC‐Heidelberg cohort as replication sets, we observed that smoking‐associated DNA methylation alterations in CpGs in the *AHRR* and *F2RL3* genes are associated with lung cancer risk^13^; the association with alterations in DNA methylation in *F2RL3* was also reported in a recent German study^14^. Our study provided initial suggestive biological plausibility and statistical evidence that these epigenetic alterations may partly mediate the effect of smoking on lung cancer risk^13^.

In order to identify novel DNA methylation changes associated with lung cancer risk and better understand the mechanisms underlying these associations, we conducted a further analysis of the four epigenome‐wide association studies (EWAS) in NOWAC, MCCS, NSHDS, EPIC‐Heidelberg and in a new, independent EWAS in the EPIC‐Italy cohort that became available recently. In all these five case‐control studies nested within prospective cohorts, we investigated associations between methylation of DNA from pre‐diagnostic, peripheral blood samples and lung cancer risk accounting for reported smoking habits.

## Methods

### Discovery and replication sets

To test our hypotheses regarding the relationship between DNA methylation and lung cancer risk, we used data from a new EWAS within the Italian component of the European Prospective Investigation into Cancer^15^ (EPIC‐Italy)^16^ and a previous one within the Melbourne Collaborative Cohort Study (MCCS)^17^ as discovery sets and data from previous EWAS from the Norwegian Women and Cancer study (NOWAC)^13^, the Northern Sweden Health and Disease Study (NSHDS)^18^ and EPIC‐Heidelberg^19^ as replication sets. All five studies were case–control studies nested within prospective cohorts including 367, 185, 132, 234, and 63 case–control pairs respectively for which methylation was measured using the Illumina Infinium Human Methylation 450 BeadChip on DNA extracted from prediagnostic, peripheral blood samples. Relative to our previous report^13^, here we present the new study within EPIC‐Italy as well as the complete data from the MCCS that in the previous report was used only to replicate the signals in the *AHRR* and *F2RL3* genes. For all studies one control was individually matched to each case. In MCCS and NSHDS controls were matched to cases by reported smoking status at blood draw using five categories (never smokers; short‐term former smokers: quitting smoking <10 years before; long‐term former smokers: quitting smoking 10 years or more before; current light smokers: <15 cigarettes per day; and current heavy smokers: 15 cigarettes or more) in EPIC‐Heidelberg they were matched by reported smoking status in two categories (current and former) and number of pack‐years. In EPIC‐Italy and NOWAC controls were not matched by smoking. Further details for each study are provided in the Supplementary Materials.

### Laboratory methods, data pre‐processing and quality control

Laboratory methods for DNA extraction, quality control, bisulphite conversion and Illumina Infinium HumanMethylation450 BeadChip assays as well as details about data pre‐processing and quality control are described in detail in the Supplementary Materials and were broadly similar across studies. Exceptions are noted explicitly. For the MCCS some DNA samples were extracted from dried blood spots on Guthrie cards using a method developed in‐house^20^. Cases with DNA available only from dried blood spots were matched to controls with the same type of DNA available.

Normalisation procedures of the methylation measures were applied to perform colour channel and probe type correction as described in the Supplementary Materials.

### Statistical analysis

For all analyses we used *M*‐values of methylation calculated as log_2_(beta/(1‐beta))^21^. To quantify the association between the methylation level at each CpG and the risk of lung cancer we fitted conditional logistic regression models separately for the two discovery sets, the MCCS and EPIC‐Italy. For EPIC‐Italy, for which smoking was not a matching variable, we adjusted the regression models for smoking. In the regression models, we included as a predictor the pseudo‐continuous *M*‐value of methylation at each CpG that we obtained by dividing the *M*‐values into quartiles according to the distribution in the control group and assigning to each category the within‐quartile median value. We estimated odds ratios per 1 standard deviation (SD) of the pseudo‐continuous variable and the corresponding 95% confidence intervals (95% CI).

We ranked the CpGs according to the p‐values of the corresponding ORs, separately for MCCS and EPIC‐Italy, and identified 34 CpGs with a *p* values lower than 10^−4^ for at least one study. For these CpGs we calculated pooled MCCS and EPIC‐Italy estimates and selected for further analyses and replication 6 CpGs whose combined estimate had a *p* values lower than 10^−5^. For the selected 6 CpGs and for the previously identified cg03636183 in the *F2RL3* gene, we estimated the ORs for lung cancer separately for MCCS, EPIC‐Italy and the three replication studies. For this set of seven CpGs, we estimated pooled ORs by combining the study‐specific estimates fitting fixed effect models overall and for different categories of smoking (never, former and current). We adjusted the estimates for current and former smokers for number of cigarettes and duration of smoking and the estimates for former smokers also for time since quitting smoking. We estimated ORs for different categories of time to diagnosis and used a likelihood ratio test to test for heterogeneity. We assessed the possible effect of cell composition on the results by adding into the models the proportions of different cell types (CD8+, CD4+, natural killer cells, B‐cells, monocytes, granulocytes) calculated using the method suggested by Houseman^22^.

We also estimated the association between *M*‐values of methylation and reported smoking for the control group by fitting a linear mixed effect model with slide (i.e. chip) nested within plate fitted as random effects and gender, age at blood collection and smoking fitted as fixed effects. We used likelihood ratio tests to test the association between methylation and smoking.

We evaluated separately for the MCCS and EPIC‐Italy the additional contribution of DNA methylation at the CpGs associated with lung cancer risk to the ability of the model to discriminate between cases and controls using area under the curve (AUC) statistics obtained from unconditional logistic regression models adjusted for the matching variables. We accounted for the contribution of smoking by including among the covariates smoking status and the number of pack‐years of cigarettes smoked.

Finally, for the CpGs whose pooled estimates had an OR with a *p* values of <10^−7^, we investigated graphically the association between DNA methylation and lung cancer risk in the 100 kilobase region around each CpG site, by plotting the pooled MCCS and EPIC ORs versus the CpG location.

## Results

### Genome‐wide association analysis in the two discovery sets

Relative to the total number of CpGs investigated across the genome, the proportion of CpGs with methylation levels inversely associated with lung cancer risk was 55% in the new EPIC‐Italy study and 53% in MCCS. Overall, for 48% of the CpGs we observed concordant associations between methylation level and lung cancer risk in MCCS and EPIC‐Italy (either both negative or both positive) and of these 58% were concordant negative.

We identified 34 CpGs for which the smoking‐adjusted association with lung cancer risk corresponds to a *p* values lower than 10^−4^ in at least one of the two studies (Supporting Information Fig. 1), and these are presented in Table 1 with their ORs and p‐values. Of these CpGs, 22 were from MCCS, 9 from EPIC‐Italy and 3 were common to both studies: cg21566642 on chromosome 2, cg05575921 in the *AHRR* gene on chromosome 5 and cg06126421 on chromosome 6. Table 1 also presents the estimates for cg03636183 in the gene *F2RL3* on chromosome 19, that, together with cg05575921 in the *AHRR* gene we previously reported to be associated with lung cancer risk^13^.

**Table 1:** “Top CpGs” in the two case‐control studies nested within the EPIC‐Italy and MCCS cohorts (discovery sets)

		UCSC_REFGENE		EPIC‐Italy (185 case‐control pairs)			MCCS (367 case‐control pairs)			Pooled estimate	
TargetID^a^	MAPINFO		CHR	OR (95% CI)	*p* values	rank	OR (95% CI)	*p* values	Rank	OR (95% CI)	*p* values
cg03256938	2983926	FLJ42875	1	0.93 (0.68‐1.27)	6.56E‐01	313561	1.59 (1.29‐1.97)	1.65E‐05	5	1.35 (1.13‐1.6)	9.04E‐04
cg18146737	92946700	GFI1	1	0.38 (0.25‐0.57)	4.35E‐06	2	0.85 (0.71‐1.02)	8.33E‐02	37354	0.75 (0.64‐0.88)	6.34E‐04
cg05951221^b^	233284402		2	0.4 (0.27‐0.61)	1.89E‐05	6	0.69 (0.55‐0.86)	9.01E‐04	264	0.61 (0.5‐0.74)	7.74E‐07
cg21566642^b^	233284661		2	0.45 (0.32‐0.64)	9.97E‐06	3	0.61 (0.48‐0.77)	3.88E‐05	15	0.55 (0.45‐0.67)	3.93E‐09
cg12749468	183755507	HTR3D	3	–	–	–	0.61 (0.48‐0.77)	3.80E‐05	12	0.61 (0.48‐0.77)	3.80E‐05
cg10663765	194014592		3	0.85 (0.58‐1.24)	3.86E‐01	192168	0.67 (0.56‐0.8)	1.87E‐05	6	0.7 (0.59‐0.83)	2.35E‐05
cg02901723	91049728	FAM190A	4	2.87 (1.74‐4.72)	3.58E‐05	8	1.07 (0.86‐1.34)	5.45E‐01	262091	1.27 (1.03‐1.55)	2.46E‐02
cg23916896	368804	AHRR	5	0.49 (0.35‐0.68)	1.79E‐05	5	0.84 (0.7‐1.01)	6.61E‐02	29301	0.73 (0.62‐0.86)	1.92E‐04
cg05575921^b^	373378	AHRR	5	0.41 (0.29‐0.57)	2.48E‐07	1	0.61 (0.48‐0.78)	8.49E‐05	20	0.53 (0.43‐0.65)	5.26E‐10
cg26853442	161178531		5	1.05 (0.79‐1.4)	7.29E‐01	346039	0.64 (0.52‐0.79)	3.12E‐05	10	0.76 (0.64‐0.9)	1.53E‐03
cg01882991	6677756		6	0.44 (0.3‐0.63)	1.12E‐05	4	0.92 (0.75‐1.12)	4.10E‐01	196120	0.77 (0.65‐0.92)	4.44E‐03
cg06126421^b^	30720080		6	0.48 (0.34‐0.68)	4.49E‐05	10	0.61 (0.49‐0.75)	2.32E‐06	1	0.57 (0.48‐0.68)	8.46E‐10
cg20421191	33217620		6	0.95 (0.66‐1.36)	7.77E‐01	367218	1.58 (1.26‐1.99)	8.87E‐05	21	1.37 (1.13‐1.66)	1.62E‐03
cg21984374	166290219		6	0.96 (0.71‐1.29)	7.66E‐01	362359	0.67 (0.55‐0.81)	3.29E‐05	11	0.74 (0.63‐0.87)	2.57E‐04
cg14586887	28966691		7	1.09 (0.74‐1.58)	6.71E‐01	320421	1.59 (1.28‐1.97)	2.87E‐05	8	1.45 (1.2‐1.75)	1.23E‐04
cg14371343	122097744	CADPS2	7	0.94 (0.69‐1.26)	6.60E‐01	315632	0.61 (0.48‐0.77)	3.82E‐05	13	0.72 (0.6‐0.86)	4.45E‐04
cg17528127	144332475	ZFP41	8	0.97 (0.71‐1.32)	8.57E‐01	402485	0.64 (0.51‐0.8)	9.62E‐05	25	0.74 (0.61‐0.89)	1.17E‐03
cg01843018	123813144	C5	9	0.51 (0.37‐0.72)	8.27E‐05	12	0.98 (0.79‐1.22)	8.53E‐01	413423	0.81 (0.67‐0.97)	2.07E‐02
cg17171127	130248270		11	1.03 (0.77‐1.37)	8.64E‐01	405797	0.65 (0.52‐0.8)	7.86E‐05	19	0.76 (0.64‐0.91)	2.34E‐03
cg16823042	58119992	AGAP2	12	0.79 (0.58‐1.08)	1.37E‐01	73212	0.69 (0.57‐0.83)	8.94E‐05	22	0.71 (0.6‐0.84)	3.73E‐05
		LOC100130776									
cg23387569^b^	58120011	AGAP2	12	0.82 (0.6‐1.12)	2.20E‐01	114030	0.67 (0.56‐0.8)	9.51E‐06	3	0.7 (0.6‐0.82)	8.47E‐06
		LOC100130776									
cg00873601	116044025		12	1.97 (1.43‐2.72)	3.86E‐05	9	1.01 (0.85‐1.2)	9.19E‐01	446272	1.17 (1.01‐1.37)	4.07E‐02
cg18754747	133526522	ZNF605	12	0.97 (0.72‐1.32)	8.59E‐01	403456	0.63 (0.51‐0.78)	2.87E‐05	9	0.73 (0.61‐0.87)	4.18E‐04
cg03261929	79168211		13	0.94 (0.71‐1.24)	6.57E‐01	314009	0.66 (0.54‐0.81)	6.94E‐05	18	0.75 (0.63‐0.88)	4.86E‐04
cg12312863^b^	2569967	AMDHD2	16	0.79 (0.58‐1.07)	1.21E‐01	65012	0.63 (0.52‐0.77)	4.00E‐06	2	0.67 (0.57‐0.79)	2.35E‐06
		ATP6V0C									
cg06279276	67184164	B3GNT9	16	1.23 (0.9‐1.69)	1.99E‐01	103877	0.67 (0.56‐0.81)	3.82E‐05	14	0.79 (0.67‐0.93)	4.02E‐03
cg16674433	19314408	RNF112	17	0.91 (0.64‐1.28)	5.78E‐01	278940	0.69 (0.57‐0.83)	6.31E‐05	16	0.73 (0.62‐0.86)	1.52E‐04
cg12076915	42430253		19	0.77 (0.55‐1.07)	1.17E‐01	63132	0.7 (0.58‐0.83)	9.33E‐05	24	0.71 (0.61‐0.84)	2.93E‐05
cg21727574	53098913	ZNF137	19	1.11 (0.8‐1.52)	5.35E‐01	259711	0.57 (0.44‐0.75)	6.47E‐05	17	0.76 (0.62‐0.93)	8.67E‐03
cg27657685	5594689		20	2.16 (1.47‐3.17)	8.20E‐05	11	1.02 (0.81‐1.28)	8.65E‐01	419192	1.24 (1.02‐1.5)	3.24E‐02
cg16767801	21686548	PAX1	20	2.19 (1.53‐3.13)	2.02E‐05	7	0.95 (0.79‐1.13)	5.60E‐01	269484	1.12 (0.95‐1.31)	1.72E‐01
cg04204002	43817086	TMPRSS3	21	1.05 (0.77‐1.44)	7.60E‐01	359676	1.61 (1.29‐2.01)	2.34E‐05	7	1.4 (1.17‐1.68)	2.71E‐04
cg26608032	41032667	MKL1	22	0.93 (0.68‐1.27)	6.52E‐01	312188	1.63 (1.31‐2.04)	1.35E‐05	4	1.35 (1.13‐1.62)	9.74E‐04
cg15224006	49125404	PPP1R3F	X	1.08 (0.65‐1.79)	7.74E‐01	365829	0.58 (0.45‐0.76)	8.99E‐05	23	0.67 (0.53‐0.85)	9.01E‐04
cg03636183^b^	17000585	F2RL3	19	0.59 (0.45‐0.78)	1.97E‐04	35	0.67 (0.54‐0.84)	3.63E‐04	88	0.64 (0.54‐0.76)	3.36E‐07

a CpGs with the *p* values for the association with lung cancer risk lower than 10^−4^ in at least one study. In the table are also reported the values for cg03636183 that we reported being associated with lung cancer risk in a previous report (Ref. 13).

b CpGs with *p* values < 1.00E‐05 for the combined estimate.

Of the associations corresponding to the 34 CpGs listed in Table 1, 73%; (95% CI, 54% to 86%, *p* = 0.01) were concordant in the two studies and, of these, 79% (95% CI, 0.57% to 0.92%, *p* = 0.008) were concordant negative (Supporting Information Fig. 1‐right panel).

**Figure 1:**
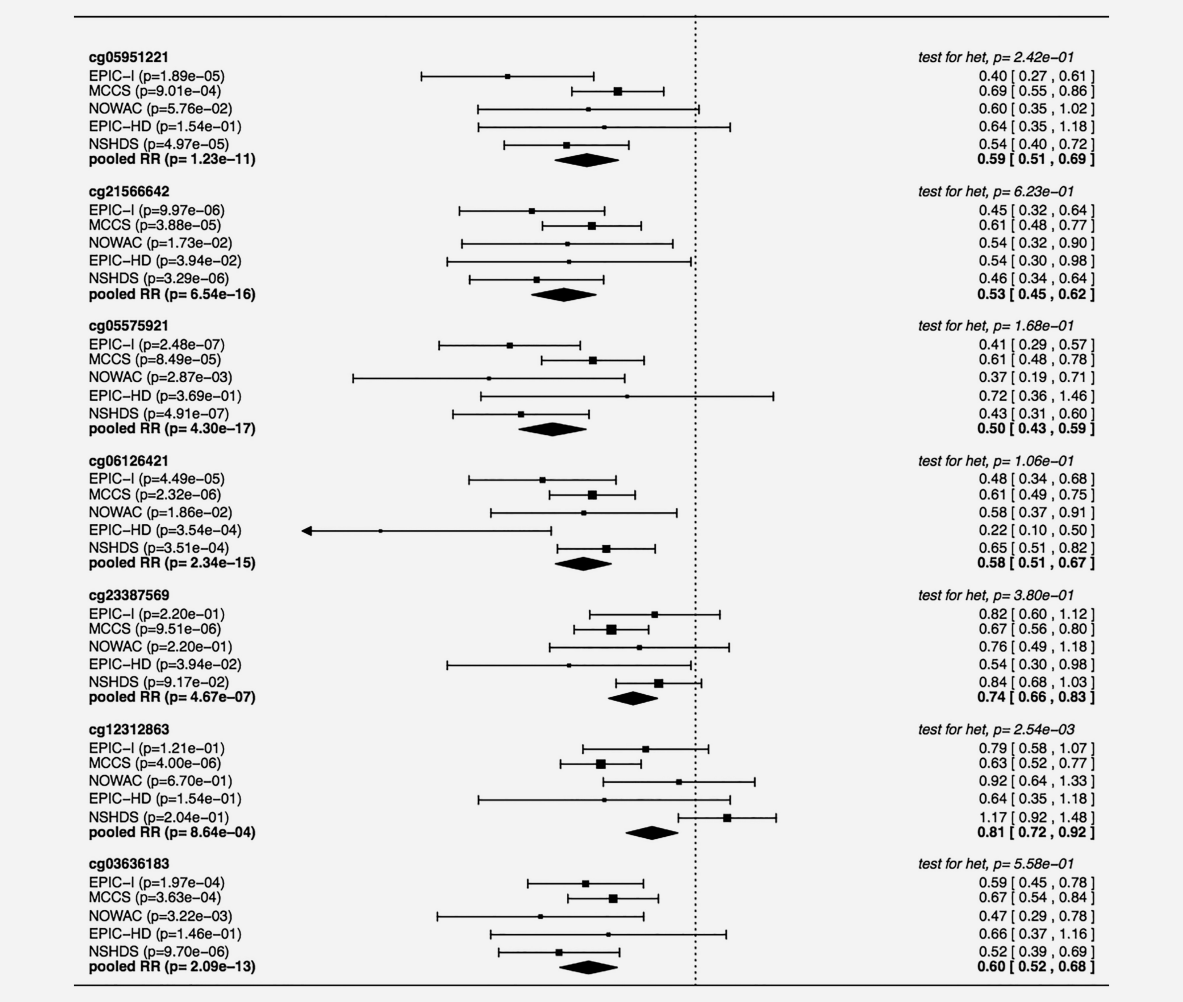
Estimated ORs for lung cancer risk for one SD increment in M methylation values separately for each of the five studies and overall (pooled estimates) for the 6 CpGs with pooled ORs across MCCS and EPIC‐Italy with *p* < 10E‐5 and cg03636183 in F2RL3.

For six CpGs (cg05951221, cg21566642, cg05575921, cg06126421, cg23387569 and cg12312863) the pooled ORs for lung cancer across EPIC‐Italy and MCCS had a *p* values lower than 10^−5^ (Table 1). For these six CpGs and for cg03636183 in the *F2RL3* gene, we tested the association with lung cancer risk in another three independent studies within NOWAC, EPIC‐Heidelberg and NSHDS (Fig. 1) by estimating the associations for each study separately and deriving pooled ORs across the five studies. For 6 of the 7 CpGs the pooled ORs had a *p* values lower than 5 × 10^−7^: cg05951221 (pooled OR per 1 SD of methylation change = 0.59; 95% CI: 0.51‐0.69, *p* = 1.23 × 10^−11^); cg21566642 (pooled OR per 1 SD, 0.53; 95% CI, 0.45‐0.62; *p*, 6.54 × 10^−16^); cg05575921 (pooled OR per 1 SD, 0.50; 95% CI, 0.43‐0.59; *p*, 4.30 × 10^−17^); cg06126421 (pooled OR per 1 SD, 0.58; 95% CI, 0.51‐0.67; *p*, 2.34 × 10^−15^); cg23387569 (pooled OR per 1 SD,0.74; 95% CI, 0.66–0.83; 4.67 × 10^−7^); cg03636183 (pooled OR per 1 SD, 0.60; 95% CI, 0.52–0.68; *p*, 2.09 × 10^−13^) (Table 2 and Fig. 1). For these six CpGs, the results did not materially change when the analyses were adjusted for estimated cell composition (Supporting Information Tables 1 and 2); we did not observe heterogeneity in the ORs for lung cancer between studies (all p‐values for heterogeneity ≥ 0.1, Fig. 1) or by time between blood draw and diagnosis overall or by smoking status (Supporting Information Table 3).

**Table 2:** Association between methylation levels and lung cancer risk in the 5 nested case‐control studies and pooled odds ratios (OR) estimated overall and by level of smoking

	All		Never		Former		Current	
	OR (95% CI)	*p*	OR (95% CI)	*p*	OR (95% CI)	*p*	OR (95% CI)	*p*
**cg05951221**								
EPIC‐Italy	0.40 (0.27‐0.61)	1.89E‐05	0.95 (0.26‐3.44)	9.36E‐01	0.02 (0.00‐1.24)	6.27E‐02	0.55 (0.09–3.38)	5.19E‐01
MCCS	0.69 (0.55‐0.86)	9.01E‐04	0.54 (0.25‐1.2)	1.30E‐01	0.59 (0.39‐0.89)	1.25E‐02	0.87 (0.62–1.21)	4.13E‐01
NOWAC	0.60 (0.35‐1.02)	5.76E‐02	1.51 (0.18‐12.76)	7.08E‐01	0.10 (0.01‐1.41)	8.74E‐02	0.52 (0.15–1.82)	3.08E‐01
EPIC‐Heidelberg	0.64 (0.35‐1.18)	1.54E‐01	–	–	0.23 (0.03‐2.03)	1.87E‐01	0.68 (0.35–1.32)	2.54E‐01
NSHDS	0.54 (0.40‐0.72)	4.97E‐05	0.85 (0.47‐1.54)	5.95E‐01	0.60 (0.35‐1.02)	5.89E‐02	0.67 (0.49–0.92)	1.29E‐02
*Pooled*	*0.59 (0.51‐0.69)*	*1.23E‐11*	*0.77 (0.5‐1.19)*	*2.40E‐01*	*0.56 (0.40‐0.77)*	*3.12E‐04*	*0.74 (0.60‐0.91)*	*5.04E‐03*
**cg21566642**								
EPIC‐Italy	0.45 (0.32‐0.64)	9.97E‐06	1.55 (0.25‐9.75)	6.38E‐01	0.31 (0.08‐1.29)	1.08E‐01	0.66 (0.22‐2.00)	4.62E‐01
MCCS	0.61 (0.48‐0.77)	3.88E‐05	0.76 (0.38‐1.51)	4.31E‐01	0.52 (0.33‐0.82)	4.91E‐03	0.72 (0.5‐1.05)	8.79E‐02
NOWAC	0.54 (0.32‐0.90)	1.73E‐02	1.1 (0.19‐6.56)	9.13E‐01	0.11 (0.01‐1.65)	1.09E‐01	0.91 (0.29‐2.82)	8.69E‐01
EPIC‐Heidelberg	0.54 (0.30‐0.98)	3.94E‐02	–	–	0.70 (0.17‐3.01)	6.35E‐01	0.49 (0.24‐0.99)	4.57E‐02
NSHDS	0.46 (0.34‐0.64)	3.29E‐06	0.83 (0.43‐1.59)	5.78E‐01	0.52 (0.28‐0.96)	3.80E‐02	0.49 (0.33‐0.73)	4.40E‐04
*Pooled*	*0.53 (0.45‐0.62)*	*6.54E‐16*	*0.84 (0.54‐1.32)*	*4.55E‐01*	*0.50 (0.36‐0.71)*	*7.47E‐05*	*0.60 (0.47‐0.77)*	*3.72E‐05*
**cg05575921**								
EPIC‐Italy	0.41 (0.29‐0.57)	2.48E‐07	0.57 (0.06‐5.73)	6.35E‐01	0.3 (0.09‐0.96)	4.22E‐02	0.69 (0.22‐2.21)	5.34E‐01
MCCS	0.61 (0.48‐0.78)	8.49E‐05	0.53 (0.18‐1.56)	2.50E‐01	0.56 (0.36‐0.87)	1.01E‐02	0.73 (0.51‐1.03)	7.71E‐02
NOWAC	0.37 (0.19‐0.71)	2.87E‐03	0.06 (0‐19.26)	3.45E‐01	–	–	0.31 (0.06‐1.51)	1.46E‐01
EPIC‐Heidelberg	0.72 (0.36‐1.46)	3.69E‐01	–	–	0.29 (0.03‐3.32)	3.23E‐01	0.70 (0.31‐1.59)	3.84E‐01
NSHDS	0.43 (0.31‐0.60)	4.91E‐07	1.66 (0.71‐3.84)	2.40E‐01	0.43 (0.24‐0.77)	4.25E‐03	0.59 (0.42‐0.82)	1.78E‐03
*Pooled*	*0.50 (0.43‐0.59)*	*4.30E‐17*	*0.99 (0.53‐1.87)*	*9.82E‐01*	*0.48 (0.35‐0.67)*	*1.74E‐05*	*0.65 (0.52‐0.81)*	*1.41E‐04*
**cg06126421**								
EPIC‐Italy	0.48 (0.34‐0.68)	4.49E‐05	0.34 (0.09‐1.29)	1.12E‐01	0.41 (0.11‐1.55)	1.90E‐01	0.92 (0.31‐2.73)	8.75E‐01
MCCS	0.61 (0.49‐0.75)	2.32E‐06	0.49 (0.21‐1.17)	1.10E‐01	0.65 (0.45‐0.93)	1.84E‐02	0.67 (0.50‐0.90)	8.09E‐03
NOWAC	0.58 (0.37‐0.91)	1.86E‐02	0.74 (0.2‐2.82)	6.62E‐01	0.50 (0.10‐2.55)	4.02E‐01	0.45 (0.13‐1.51)	1.96E‐01
EPIC‐Heidelberg	0.22 (0.10‐0.50)	3.54E‐04	–	–	0.30 (0.06‐1.43)	1.29E‐01	0.16 (0.05‐0.50)	1.76E‐03
NSHDS	0.65 (0.51‐0.82)	3.51E‐04	1.3 (0.72‐2.37)	3.87E‐01	0.56 (0.36‐0.89)	1.35E‐02	0.7 (0.51‐0.94)	1.85E‐02
*Pooled*	*0.58 (0.51‐0.67)*	*2.34E‐15*	*0.83 (0.54‐–1.28)*	*4.01E‐01*	*0.59 (0.45–0.77)*	*1.05E‐04*	*0.65 (0.53‐0.8)*	*3.20E‐05*
**cg23387569**								
EPIC‐Italy	0.82 (0.60‐1.12)	2.20E‐01	1 (0.44‐2.31)	9.96E‐01	2.97 (0.81‐10.9)	1.00E‐01	1.3 (0.38‐4.45)	6.78E‐01
MCCS	0.67 (0.56‐0.80)	9.51E‐06	0.53 (0.3‐0.96)	3.58E‐02	0.47 (0.32‐0.7)	1.93E‐04	0.79 (0.62‐1.01)	6.05E‐02
NOWAC	0.76 (0.49‐1.18)	2.20E‐01	1.18 (0.2‐7.02)	8.55E‐01	1.54 (0.17‐13.66)	7.01E‐01	0.76 (0.38‐1.55)	4.53E‐01
EPIC‐Heidelberg	0.54 (0.30‐0.98)	3.94E‐02	–	–	0.7 (0.17‐3.01)	6.35E‐01	0.49 (0.24‐0.99)	4.57E‐02
NSHDS	0.84 (0.68‐1.03)	9.17E‐02	0.87 (0.48‐1.56)	6.39E‐01	0.86 (0.57‐1.29)	4.65E‐01	0.85 (0.61‐1.17)	3.08E‐01
*Pooled*	*0.74 (0.66‐0.83)*	*4.67E‐07*	*0.75 (0.52‐1.08)*	*1.21E‐01*	*0.69 (0.52‐0.9)*	*6.31E‐03*	*0.79 (0.66‐0.94)*	*9.73E‐03*
**cg03636183**								
EPIC‐Italy	0.59 (0.45‐0.78)	1.97E‐04	0.2 (0.03‐1.42)	1.09E‐01	0.44 (0.18‐1.05)	6.48E‐02	0.48 (0.14‐1.71)	2.58E‐01
MCCS	0.67 (0.54‐0.84)	3.63E‐04	0.73 (0.38‐1.4)	3.43E‐01	0.66 (0.45‐0.97)	3.35E‐02	0.8 (0.57‐1.13)	2.08E‐01
NOWAC	0.47 (0.29‐0.78)	3.22E‐03	0.58 (0.01‐39.48)	8.03E‐01	0.25 (0.04‐1.53)	1.33E‐01	0.64 (0.24‐1.67)	3.60E‐01
EPIC‐Heidelberg	0.66 (0.37‐1.16)	1.46E‐01	–	–	0.26 (0.02‐2.92)	2.75E‐01	0.73 (0.4‐1.35)	3.15E‐01
NSHDS	0.52 (0.39‐0.69)	9.70E‐06	1.42 (0.73‐2.76)	2.96E‐01	0.41 (0.22‐0.73)	2.81E‐03	0.66 (0.46‐0.94)	2.22E‐02
*Pooled*	*0.60 (0.52‐0.68)*	*2.09E‐13*	*0.93 (0.59‐1.45)*	*7.35E‐01*	*0.54 (0.4‐0.72)*	*3.76E‐05*	*0.72 (0.58‐0.89)*	*2.95E‐03*

### Associations with reported smoking history and associations with lung cancer risk by smoking category

Of the six CpGs associated with lung cancer risk, the methylation levels of five (cg05951221, cg21566642, cg05575921, cg06126421, and cg03636183) were strongly associated with reported smoking history in the control groups (p‐values for heterogeneity across smoking categories all ≤ 1.8 × 10^−7^). DNA methylation levels were lowest for current smokers while average levels for former smokers were intermediate between those for current and never smokers; DNA methylation levels for former smokers increased with increasing time since quitting (Table 3; Supporting Information Figs. 2 and 3).

**Table 3:** Association between methylation levels and smoking status in controls from EPIC‐Italy and MCCS at the 6 CpGs associated with lung cancer risk

		EPIC‐Italy			MCCS		
Probe	Smoking	coef (95% CI)	*p*	*p‐het* a	coef (95% CI)	*p*	*p‐het* a
cg05951221	Never	Reference	‐	1.59E‐13	Reference	‐	1.18E‐21
	Former	−0.27 (−0.39, −0.16)	7.28E‐06		−0.3 (−0.44, −0.16)	3.05E‐05	
	Current	−0.54 (−0.68, −0.41)	1.23E‐13		−0.63 (−0.76, −0.49)	6.18E‐18	
cg21566642	Never	reference	‐	7.98E‐17	reference	‐	9.99E‐32
	Former	−0.34 (−0.48, −0.2)	3.00E‐06		−0.3 (−0.45, −0.14)	2.13E‐04	
	Current	−0.77 (−0.93, −0.6)	4.45E‐17		−0.82 (−0.98, −0.67)	7.73E‐23	
cg05575921	Never	reference	‐	1.98E‐15	reference	‐	1.67E‐40
	Former	−0.27 (−0.48, −0.07)	9.42E‐03		−0.24 (−0.44, −0.03)	2.29E‐02	
	Current	−1.07 (−1.3, −0.83)	7.28E‐16		−1.12 (−1.32, −0.92)	3.47E‐24	
cg06126421	Never	Reference	‐	1.81E‐07	Reference	‐	3.85E‐13
	Former	−0.23 (−0.4, −0.06)	7.96E‐03		−0.34 (−0.56, −0.12)	2.70E‐03	
	Current	−0.57 (−0.77, −0.38)	4.64E‐08		−0.74 (−0.95, −0.52)	6.19E‐11	
cg23387569	Never	Reference	‐	7.37E‐04	Reference	‐	1.23E‐01
	Former	−0.39 (−0.67, −0.11)	6.32E‐03		0.02 (−0.25, 0.29)	8.91E‐01	
	Current	−0.57 (−0.9, −0.25)	5.92E‐04		−0.15 (−0.42, 0.11)	2.65E‐01	
cg03636183	Never	Reference	‐	1.28E‐11	Reference	‐	1.18E‐23
	Former	−0.16 (−0.27, −0.04)	7.04E‐03		−0.22 (−0.38, −0.07)	4.30E‐03	
	Current	−0.5 (−0.64, −0.37)	3.18E‐12		−0.67 (−0.82, −0.52)	1.14E‐16	

a *p* values of the test of homogeneity by smoking.

To investigate whether the association between methylation levels at the 6 CpGs and lung cancer risk could be due to residual confounding by smoking, we conducted stratified analyses by smoking status separately for each of the five studies and overall (Table 2). For all the CpGs the pooled ORs were lower than unity for former and current smokers and the ORs were consistently lower for former smokers than for current smokers; for all CpGs the OR for never smokers was nominally lower than unity but none of the ORs for never smokers were statistically significant.

### Ability of methylation levels to predict lung cancer risk

For EPIC‐Italy, the value of the AUC for the model including reported smoking status (categorised as never, former and current smoker) and the number of pack years was 79%. Adding methylation levels for each CpG individually resulted in gains of 1.8% for cg05575921, 1.3% for cg06126421 and <1% for each of the other selected CpGs (Table 4 and Supporting Information Fig. 4). When methylation levels for all the 6 CpGs were included simultaneously in the model, their additional contribution to lung cancer risk prediction was 2.6% (*p* = 0.034) overall; 11.1% (*p* = 0.011) in former smokers and 1.2% in current smokers (*p* = 0.52). We obtained similar results from the MCCS in which the overall gain from including all the CpGs combined was 5.5% (*p* = 0.002) relative to the model including smoking history (categorized as never smokers; former smokers who stopped <10 years before blood draw; former smokers who stopped 10 or more years before blood draw; current smokers who smoked <15 cigarettes per day; and current smokers who smoked 15 or more cigarettes per day) and number of pack‐years; the gain was 7.6% (*p* = 0.004) in former smokers and 3.3% (*p* = 0.28) in current smokers.

**Table 4:** Contribution to the ability to predict the risk of lung cancer by methylation level in addition to smoking status and smoking intensity (pack‐years)

	cg05951221	cg21566642	cg05575921	cg06126421	cg23387569^a^	cg03636183	All^a^
**EPIC‐Italy**							
mod1∼smoke	0.718	0.718	0.718	0.718	0.721	0.718	0.721
mod2∼smoke+M	0.758	0.751	0.784	0.769	0.725	0.758	0.797
AUC_mod1_‐AUC_mod2_	0.040	0.033	0.066	0.051	0.004	0.040	0.076
*p*	0.017	0.040	0.001	0.005	0.548	0.009	*2.64E‐4*
mod5∼smoke+pckyrs	0.789	0.789	0.789	0.789	0.791	0.789	0.791
mod6∼smoke+pckyrs+M	0.794	0.795	0.807	0.802	0.795	0.797	0.817
AUC_mod3_‐AUC_mod4_	0.005	0.006	0.018	0.013	0.004	0.008	0.026
*p*	0.407	0.274	0.100	0.156	0.491	0.157	0.035
**MCCS**							
mod3∼smoke	0.491	0.491	0.491	0.491	0.491	0.491	0.491
mod4∼smoke+M	0.569	0.584	0.586	0.603	0.599	0.572	0.640
AUC_mod3_‐AUC_mod4_	0.078	0.093	0.095	0.112	0.108	0.081	0.149
*p*	0.018	0.003	0.003	*2.68E‐4*	0.001	0.011	*9.74E‐7*
mod5∼smoke+pckyrs	0.610	0.610	0.610	0.610	0.610	0.610	0.610
mod6∼smoke+pckyrs+M	0.621	0.626	0.624	0.641	0.640	0.624	0.665
AUC_mod5_‐AUC_mod6_	0.011	0.016	0.014	0.031	0.030	0.014	0.055
*p*	0.315	0.205	0.232	0.038	0.048	0.234	0.002

All statistics have been calculated from logistic regression models adjusted for the matching variables specific to the study (see method section for details). In EPIC‐Italy smoking status is coded never, former and current. In MCCS it is coded never; former who stopped <10 years before blood draw; former who stopped 10 or more years before blood draw; current smokers who smoked <15 cigarettes per day; current smokers who smoked 15 or more cigarettes per day.

a From the analyses including cg23387569 we have excluded two samples with missing values in the methylation level of this CpG.

Abbreviations: AUC, area under the curve.

### Analyses of regions around the CpGs associated with lung cancer risk

**Figure 2:**
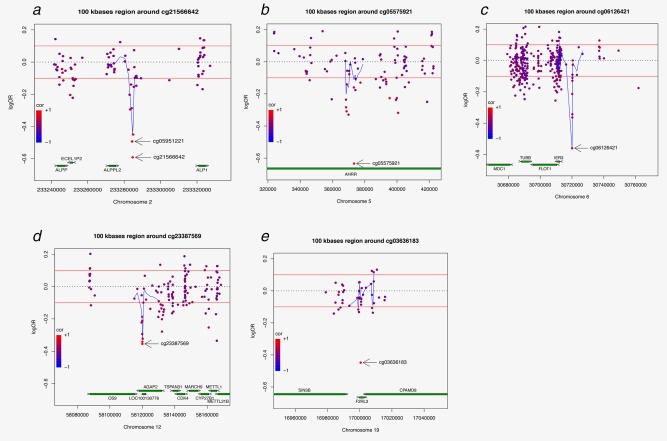
Association between M methylation levels and lung cancer risk in the regions (100k bases) around the CpGs associated with lung cancer risk (panels *a*–*e* represent regions in 2q37.1, 5p15.33, 6p21.33 and 12q14.1, respectively including the CpGs associated with lung cancer risk).

Both cg05951221 and cg21566642 are located in a CpG island on chromosome 2q37.1 in which a region with differential methylation between cases and controls is clearly visible (Fig. 2a); this region extends for approximately 2 kilobases and includes 8 CpGs for which lower methylation levels are associated with an increased risk of lung cancer. The correlation between the methylation M‐values for cg05951221 and cg21566642 (259 bases apart) was 0.80 and for cg21566642 and cg01940273 (273 bases apart) was 0.75. The 100k‐base region around cg05951221 and cg21566642 contains three genes (*ALPPL2, ALPP,* and *ALP‐1, and ECEL1*) and the pseudogene *ECEL1P2* whose methylation has been found to increase through development^23^. Alkaline phosphatases (ALPs) dephosphorylate a variety of molecules such as proteins, nucleotides and alkaloids. Serum ALPP and ALPPL2 enzyme levels are increased in heavy smokers and in cancer, particularly in seminoma^24^.

We observed no significant correlation between cg05575921, located within the *AHRR* gene on chromosome 5p15.33, and the nearby CpGs (Fig. 2b). In the 100 kilobases flanking the probe maps one more gene (*EXOC3*) that codes for a component of the exocyst complex and its antisense RNA (*EXOC3‐AS1*).

The probe cg06126421 on chromosome 6p21.33 is flanked by a region extending approximately 200 bases containing another 5 CpGs whose methylation levels correlate with methylation levels of cg06126421 (correlations ranging from 0.44 to 0.67) (Fig. 2c). In the 100 kilobases flanking cg06126421 there are seven genes that code for proteins involved in cell cycle checkpoints in response to DNA damage (*MDC1*), cellular growth and division (*DHX16*), protection of cells from Fas‐ or tumor necrosis factor type alpha‐induced apoptosis (*IER3*), cytoskeleton regulation and membrane traffic (*PPP1R18*, *TUBB, FLOT1, NRM)*. In particular, *FLOT1* mRNA expression has been shown to be upregulated in non‐small cell lung cancer tissue^25^. Also, two long non‐coding RNAs (*lncRNAs*) map in the region: *MDC1‐AS1* and *LINC00243*.

A region of ∼300 bases extends around cg23387569 on chromosome 12q14.1 as methylation levels for the six CpG sites in this region quite strongly correlate with methylation level at cg23387569 (correlations ranging from 0.52 to 0.87) (Fig. 2d). The region is located in a CpG island within the *AGAP2* gene, which encodes a protein belonging to the centaurin gamma‐like family that mediates anti‐apoptotic effects of nerve growth factor by activating nuclear phosphoinositide 3‐kinase. The *AGAP2* gene is overexpressed in cancer cells, and promotes cancer cell invasion. The region surrounding cg23387569 has been previously found amplified in lung cancer^26^ together with other four genes that map in the region (*CDK4*, *CYP27B1*, *METTL1*, and *TSFM*). One of them codes for the cyclin‐dependent kinase 4, a member of the Ser/Thr protein kinase family that is important for cell cycle G1–S transition by the RB1–CCND1–CDKN2A pathway that is known to be damaged in lung cancer.

Other genes in the 100‐kilobase region include *AVIL*, *B4GALNT1*, *OS9*, *TSPAN31*, *MARCH9*, *METTL21B, TSPAN31* and two miRNAs: MIR6759 and MIR26A2

We observed no correlation between cg03636183, located within the *F2RL3* gene on chromosome 19p13.11, and the nearby CpGs (Fig. 2e).

## Discussion

This new analysis, combining data from the four EWAS that previously allowed us to identify the first two CpGs in *AHRR* and *F2RL3* associated with lung cancer risk with previously unpublished new data from a novel EWAS in EPIC‐Italy, led to the discovery of four additional CpGs and showed that methylation at these CpGs may be useful to improve current risk prediction models based on self‐reported smoking history.

Our previous report that DNA methylation changes at cg05575921 in the *AHRR* gene and at cg03636183 in the *F2RL3* gene were associated with lung cancer risk^13^ included mediation analyses which provided initial suggestive evidence that residual confounding was unlikely to explain the observed associations for cg05575921 and cg03636183, and that hypomethylation at these two sites may mediate the effect of tobacco on lung cancer risk. For cg03636183, an association with lung cancer risk similar to the one we observed was also reported in a study of 4,987 participants in the German ESTHER cohort, of which 97 developed lung cancer during a median follow‐up of around 11 years^14^. In ESTHER only three CpGs in the *F2RL3* gene, including cg03636183, were measured using mass‐spectrometry (i.e. MALDI‐TOF) and were targeted because of the established strong association between cigarette smoking and methylation at this site.

Relative to our previous report^13^, in the present analyses we have included data from a new case‐control study nested within EPIC‐Italy and presented the complete data from the MCCS that was previously utilised only to validate the CpGs within the *AHRR* and *F2RL3* genes; consequently, the results reported here for all other CpGs can be considered original and independent from those previously published. To investigate the possible role of smoking in explaining the observed associations between DNA methylation and lung cancer risk, we deliberately oversampled cases of former and never smokers from some of the cohorts.

The associations we observed between DNA methylation and lung cancer risk are relatively strong (ORs for 1 SD increase in DNA methylation are between 0.74 and 0.50), they are not limited to current smokers and they remained strong after adjusting for smoking duration and intensity: this suggests that the associations between DNA methylation and lung cancer risk are unlikely to be explained by residual confounding by smoking. The observation that for all the identified CpGs except one the methylation levels are lower in current smokers and rise to the levels of never smokers with increasing time since quitting suggests that smoking contributes to the methylation status of these CpGs, although it might not be the only determinant and disentangling the relation between smoking, methylation and lung cancer risk might be challenging^27^.

Interestingly, for all the six CpGs identified the ORs for lung cancer for former smokers are consistently lower than ORs for current smokers. This observation, consistent across all five studies, is intriguing but difficult to explain. The analyses by smoking have been adjusted for smoking intensity, duration and time since quitting in former smokers, but we cannot exclude that the result is due to misreported smoking habits or residual confounding. It has been recently reported that inflammation processes such as those caused by smoking induce changes in the methylation profile of natural killer cells including hypomethylation in the *AHRR* gene^28^ for which we observe associations with smoking and lung cancer risk in our studies. The stronger associations observed in former smokers might reflect the activation of a persistent immune response to smoking that continues and does not resolve years after smoking cessation for selected ex‐smokers who develop lung cancer. Although the adjustment for cell composition with the algorithm proposed by Houseman and colleagues does not materially modify any of the observed associations, the algorithm does not include all minor immune cell fractions that might still have a role in confounding the results^22^.

Lung diseases, including lung cancer, may trigger an immune response and alter the prevalence of specific cell types in the blood^29^; it is therefore possible that the immune response generated by undiagnosed lung cancer already present for some cases at baseline may lead to differences in the overall methylation profile that could potentially explain our findings. However, the possibility that the observed associations are because of the effect of subclinical lung cancer is not supported by our data as the observed associations did not change when the analyses were stratified by time between blood draw and lung cancer diagnosis.

The observations that at cg23387569 the association between methylation levels and smoking history was not evident or at least not as strong as for the other CpGs and that for all CpGs the associations between DNA methylation levels and lung cancer were not limited to current smokers suggest that DNA methylation changes at these CpGs may play a role in pathways to lung cancer that are independent of smoking. Further studies specifically designed to increase the number of lung cancer cases in never smokers are necessary to provide convincing evidence to support this hypothesis.

The use of the Illumina Infinium HumanMethylation 450 BeadChip allowed us to obtain epigenome‐wide data at single CpG resolution for a relatively large number of cases and controls but these microarray data do not permit the systematic investigation of the regions surrounding the CpGs identified to evaluate whether the observed associations may be regional and thus have greater predictive power should a more comprehensive measure of methylation be possible. In the region on chromosome 2q.37.1, for example, two measured CpGs (cg21566642 and cg05951221) were both strongly associated with lung cancer risk and others in the same region show suggestive evidence of association. It is possible that methylation at other unmeasured sites in this region might be even more strongly associated with lung cancer risk. It is, therefore, important that further studies, for example based on targeted bisulphite sequencing, are conducted to finely map methylation in these regions.

To further investigate the functional relevance of the observed associations it would be important to test whether methylation in the CpGs identified alter the expression of proximal genes. We could not do this directly in our cohorts as we do not have gene expression data but we have investigated it in other datasets and available public data. In a previous study we showed that methylation at cg05575921 was associated with decreased expression of the *AHRR* gene both in lung tumour tissue from current smokers and in mouse models of exposure to cigarette smoking^7^.

For the three probes that map within a gene sequence (cg05575921 in *AHRR*; cg23387569 in *AGAP2*; cg03636183 in *F2RL3*) we investigated the correlation between methylation and expression using TCGA (http://cancergenome.nih.gov/) and HapMap (http://hapmap.ncbi.nlm.nih.gov/) data^13^. In the latter, only data for the *F2RL3*‐probe were available. In brief, *AHRR‐*probe methylation seems to be inversely correlated with *AHRR* expression in lung tumour tissue from TCGA. *F2RL3‐*probe methylation does not show methylation‐expression correlation in TCGA data but HapMap data suggest a weak inverse correlation (Pearson's correlation coefficient = −0.28, *p* value < 0.01). *AGAP2*‐probe methylation seems to be positively correlated with *AGAP2* expression in both lung tumour tissue from adenocarcinomas and squamous cell carcinomas (Pearson's correlation coefficient = 0.49, *p* value 0.025; and Pearson's correlation coefficient = 0.55, *p* value = 0.15 respectively).

In the study within the ESTHER cohort, the authors estimated that the gain in ability to discriminate between cases and controls by adding methylation levels in cg03636183 in the *F2RL3* gene to a model that included smoking with pack‐years smoked was marginal (<1% increase in the AUC or C‐statistics)^14^. The analysis we conducted showed that the inclusion of the methylation level of all 6 CpGs in the prediction model produced an overall gain between 3% and 6% in its discriminatory ability; the gain was as high as 8% to 13% in former smokers. These findings encourage further work to increase the sample size and genome coverage to identify further regions with altered DNA methylation associated with lung cancer risk and use this new information to improve current risk prediction models for lung cancer especially in former smokers and test the new models in terms of both their ability to discriminate between cases and controls and the accuracy of the predicted probabilities.

## Supporting information

Supporting Information

Supporting Figure Legend

Supporting Figure 1

Supporting Figure 2

Supporting Figure 3

Supporting Figure 4

Supporting Table 1

Supporting Table 2

Supporting Table 3
