# Supplementary material for: DNA methylation changes measured in pre‐diagnostic peripheral blood samples are associated with smoking and lung cancer risk
Source: Int J Cancer. 2016 Oct 11;140(1):50–61. doi: 10.1002/ijc.30431 (PMC5731426; doi:10.1002/ijc.30431)
Supplement: Supplementary file 1 — Supporting Information [file IJC-140-50-s001.docx]

**DNA methylation changes measured in pre-diagnostic peripheral blood samples are associated with smoking and lung cancer risk**

**SUPPLEMENTARY MATERIALS**

**STUDY POPULATIONS**

To test our hypotheses regarding the relationship between DNA methylation and lung cancer risk, we used data from two epigenome-wide association studies from the Melbourne Collaborative Cohort Study (MCCS) and from the Italian component of the European Prospective Investigation into Cancer (EPIC-Italy) as discovery sets and data from the Norwegian Women and Cancer study (NOWAC), EPIC-Heidelberg, and the Northern Sweden Health and Disease Study (NSHDS) as replication sets. All five studies were case-control studies nested within prospective cohorts that measured DNA methylation in prediagnostic, peripheral blood samples using the Illumina Infinium Human Methylation 450 BeadChip.

**Discovery sets**

*MCCS*

The MCCS is a prospective cohort study of 41,514 volunteers (24,469 women) aged between 27 and 76 years at baseline (99.3% of whom were aged 40-69). At baseline attendance in 1990-1994, participants completed questionnaires that measured demographic characteristics and lifestyle factors. Height and weight were directly measured and a blood sample was collected and stored. For 25% of participants, buffy coat or lymphocyte samples were collected and stored in liquid nitrogen, while for the rest of the cohort only dried blood spots on Guthrie cards were stored. A total of 533 incident cases of lung cancer identified through linkage with the State and National Cancer Registries were diagnosed during follow-up up to the end of 2011. A total of 367 cases remained available after excluding cases 1) diagnosed after the age of 80 years; 2) with no biospecimen available; 3) with a diagnosis of any cancer before blood draw; or 4) with no information on smoking status. The MCCS sample included 367 cases (159 adenocarcinomas, 33 large cell cancers, 73 squamous cancers and 49 small cell cancers) and 367 matched controls selected from MCCS participants who were lung cancer free at the age of diagnosis of the matching case (density sampling). Matching variables included gender, date of blood collection (within 6 months), date of birth (within 1 year), country of birth (Australia and UK versus Southern Europe), type of biospecimen (lymphocyte, buffy coat and dried blood spot) and smoking status (never smokers; short-term former smokers: quitting smoking less than 10 years before blood draw; long-term former smokers: quitting smoking 10 years or more before blood draw; current light smokers: less than 15 cigarettes per day at blood draw; and current heavy smokers: 15 cigarettes or more at blood draw). In the study sample, the mean time between blood draw and diagnosis was 9.38 years (SD, 5 years).

*EPIC-Italy*

EPIC-Italy is the Italian component of EPIC, a large multicenter European prospective cohort study on diet and chronic diseases. EPIC-Italy includes 47,749 volunteers (32,579 women) aged 35–70 years at the time of recruitment 1992–1998. Anthropometric measurements and lifestyle variables including detailed information on smoking and smoking history were collected at recruitment through standardized questionnaires, together with a blood sample that was sent to local laboratories for processing and aliquot preparation. Blood was separated into 0.5 mL fractions and stored in liquid nitrogen at −196°C. All participants signed an informed consent form, and the ethical review boards of the International Agency for Research on Cancer and of each local participating centre approved the study protocol.

Within EPIC-Italy we conducted a nested case-control study utilizing 192 incident cases diagnosed within follow-up and 192 healthy controls individually matched to cases by gender, date of birth (±5 years), date of inclusion in the study and study centre. Four case-control pairs have been excluded form the study because one of the two samples didn’t pass the quality control check, three pairs were excluded as they had missing information on smoking history.

Within EPIC-Italy we conducted a nested case-control study utilizing 192 incident cases diagnosed within follow-up and 192 controls individually matched to cases by gender, date of birth (±5 years), date of inclusion in the study and study centre. Four case-control pairs have been excluded from the study because one of the two samples didn’t pass the quality control check, three pairs were excluded as they had missing information on smoking history

**Replication sets**

*NOWAC*

The biobank of the NOWAC cohort was established in the years 2003-2006. Random samples of Norwegian women were mailed a letter of information with an invitation to receive equipment for blood sampling at the local doctor or other institutions. Those who filled in an eight-page questionnaire and accepted the invitation to donate blood were sent blood drawing equipment together with a two-page epidemiological questionnaire. Around 50 000 women returned two tubes of blood by over-night mail to the Institute of Community Medicine at UiT The Artic University of Norway. Upon arrival, the citrate glass tube was centrifuged and buffy-coat and plasma frozen immediately at –80 degrees together with a PAXgene tube. All participants gave informed consent. The study was approved by the Regional Committee for Medical and Health Research Ethics in North Norway. Data storage and linkage to the National Cancer Registry of Norway was approved by the Norwegian Data Inspectorate. During follow-up to the end of 2011, 132 eligible cases of lung cancer were identified and were used for the methylome-wide association study. For each case, one control with an available blood sample was selected and matched on time since blood sampling and year of birth in order to control for effects of storage time and ageing. The cases and the controls were processed together for all laboratory procedures in order to reduce any batch effect.

*NSHDS*

NSHDS is an ongoing prospective cohort and intervention study intended for health promotion of the population of Västerbotten County in northern Sweden. Study participants were recruited to the NSHDS in the context of the Västerbotten Intervention Project (VIP), which was initiated in 1985 to advocate a healthy diet and lifestyle. All residents in Västerbotten County were invited to participate by attending a health check-up at their local health care centre at 40, 50 and 60 years of age. At the health check-up, participants were asked to complete a self-administered questionnaire covering various factors such as education, smoking habits, physical activity and diet. In addition, height and weight were measured and participants were asked to donate a blood sample of 20mL for future research. Incident lung cancer cases were identified through linkage to the regional cancer registry. Lung cancer cases were defined on the basis of the International Classification of Diseases for Oncology, Second Edition (ICD-O-2), and included all primary malignant cancers coded as C34.0-C34.9 with pre-diagnostic blood samples. One control was chosen at random for each lung cancer case from appropriate risk sets consisting of all cohort members alive and free of cancer (except non-melanoma skin cancer) at the time of diagnosis of the index case. Matching criteria were the same as for the MCCS except there was no matching for type of biospecimens as DNA was extracted from whole blood for all samples. After quality control, a total of 234 incident lung cancer cases (111 adenocarcinomas, 6 large cell cancers, 47 squamous cancers, and 29 small cell cancers) and 234 individually matched controls were available for this analysis. For the sample, the mean time from blood draw to diagnosis was 9.6 years (range: 1.1-17.5).

*EPIC-Heidelberg*

EPIC-Heidelberg is a prospective cohort study and one of the components of EPIC. It includes 25,500 participants recruited from the general population between June 1994 and October 1998. Residents of the city of Heidelberg, Germany, and its surrounding region who met the age criteria of the EPIC study design (men: 40–64, women: 35–64) were randomly invited by mail to take part in the study. Study subjects were asked to complete questionnaires and were interviewed about their individual health, diet and lifestyle such as life history of tobacco smoking and alcohol intake. Additionally, anthropometric measurements were taken and a blood sample of 30ml was collected which was aliquoted and stored in liquid nitrogen for future research. Up to six follow up questionnaires were sent to the participants, at 2 to 3-year intervals, to ask about incident diseases and changes in lifestyle and diet. All self-reported incident cases of cancer were systematically verified against clinical and pathology records. The present study was based on 211 incident lung cancer cases identified by July 2015. Cases with less than one year from blood draw to diagnosis were excluded. Of the remaining cases those with the shortest follow-up times to diagnosis and who were either current or former smokers at the baseline recruitment were selected for this study (n=66). Controls without any neoplastic disease were randomly matched to the lung cancer cases using a density sampling protocol. Matching was done on the basis of age at baseline (± 5 years), gender, smoking status (current and former), and number of pack years (± 1). After initial quality control 63 incident lung cancer cases (25 adenocarcinomas, 15 squamous cell carcinoma, 19 small cell lung cancer and 4 uncharacterized lung cancers) with a mean interval between blood draw and diagnosis of 4.8 years (range: 1.1-8.6) and 63 individually matched controls remained for further analysis. The study was approved by the Ethics Committee of the Medical Faculty of the University of Heidelberg (S-627/2013).

**Laboratory methods**

For the MCCS, laboratory procedures were carried out at the Genetic Epidemiology Laboratory, the University of Melbourne according to manufacturers' protocols. DNA extraction from lymphocytes and buffy coats was performed using Qiagen mini spin columns (Hilden, Germany) while dried blood spot DNA was extracted using a method developed in-house and the quality and quantity of DNA was assessed using the Quant-iT™ Picogreen® dsDNA assay measured on the Qubit® Fluorometer (Life Technologies, Grand Island, NY). Samples were distributed into 96-well plates and processed in chips of 12 arrays (8 chips per plate) with case-control pairs arranged randomly on the same chip. 500 ng of DNA were bisulphite-converted using the Zymo Research EZ-96 DNA Methylation-Gold™ Kit, and hybridised to Illumina Infinium HumanMethylation450 BeadChip. These were subsequently scanned using the Illumina HiScanSQ system, and sample quality was assessed using control probes present on the micro-arrays. Finally, raw intensity data were exported from Illumina GenomeStudio (version 2011.1).

For NOWAC and EPIC-Italy the laboratory procedures were carried out at the Human Genetics Foundation (Turin, Italy). Buffy coats stored in liquid nitrogen were thawed, and genomic DNA was extracted using the QIAGEN QIAsymphony DNA Midi Kit. All subsequent steps were performed as described above for the MCCS.

Laboratory procedures for NSHDS were carried out at two sites. DNA extraction from the buffy coat of EDTA-venous blood samples was conducted at Umeå University, Sweden, using FlexiGene DNA Kit (QIAGEN GmbH, Hilden, Germany). Illumina Infinium HumanMethylation450 BeadChip analysis was conducted at the ALSPAC/IEU Laboratory at the University of Bristol, according to the protocol described above for the MCCS.

EPIC-Heidelberg laboratory procedures were carried out at LGC Bioscience (United Kingdom) and at the German Cancer Research Center (DKFZ; Heidelberg, Germany). Buffy coat DNA was isolated at LGC Bioscience by the company’s standardized protocols and returned to DKFZ. DNA methylation profiling with the Illumina Infinium HumanMethylation450 BeadChip array was performed according to the manufacturer’s instructions at the DKFZ Genomics and Proteomics Core Facility. Quality control of genomic DNA included three independent measurements with Quant-iT™ Picogreen® dsDNA assay and all samples were tested on 1% agarose gels for DNA integrity. All subsequent steps were performed as described for the MCCS.

*Data pre-processing and quality control*

For the MCCS, methylation data were normalized to the internal built-in controls as provided by the standard Illumina software and subset-quantile within array normalization (SWAN) for type I and II probe bias correction. The 65 CpGs corresponding to single nucleotide polymorphisms were excluded. Methylation measures were assigned as missing for CpG sites with a detection p-value higher than 0.01. No samples failed (a sample was considered as "failed" if more than 5% of the CpG measures were missing) and 182 (0.04%) CpG sites where excluded because values were missing for more than 20% of the samples, thus leaving 485,330 CpGs suitable for the analysis. Only the 458 male samples were considered when filtering probes in the Y chromosome.

For NOWAC and EPIC-Italy data pre-processing was carried out using in-house software written for the R statistical computing environment. For each sample and each probe, measurements were set to missing if obtained by averaging intensities over less than three beads, or if averaged intensities were below detection thresholds estimated from negative control probes. Background subtraction (to remove background noise) and dye bias correction (for probes using the Infinium II design) were also performed. The resulting subset of 473,929 probes targeting autosomal CpG loci was selected for further analyses, and among these, probes with missing values in more than 20% of the samples were excluded from the analyses, leaving 450,890 probes. Samples with more than 5% of non-detected probes were also excluded from the analysis (14 samples excluded).

For the NSHDS, methylation data were normalized using a functional normalization procedure that uses the built-in control probes to remove unwanted technical variation. CpG sites that mapped to multiple genomic regions were excluded. CpG sites with a detection p-value >0.01 were set to missing. CpG sites were excluded if they were missing in more than 20% of samples. Samples were excluded if more than 5% of their CpG sites were missing or if their average detection p-value was >0.01. Samples were also dropped if their case-control pair was missing. Of 490 samples initially available, 22 were excluded on the basis of the aforementioned procedures, leaving a total of 234 matched case-control pairs for analysis.

For EPIC-Heidelberg, the quality control measures included removal of SNP-containing probes, removal of CpGs not analysed in all samples or those in non-CpG context, correction for batch effects and normalization with beta quantile dilation method: 63 sample pairs entered the final differential methylation analysis.

**LEGENDS to Supplementary Figures**

**Supplementary Figure 1** Results of the MWAS in MCCS and EPIC-Italy. (Left panel) Manhattan plot of the log(OR) of the association between lung cancer risk and a 1 SD increment in the methylation level of each CpG. (Right panel) Comparison between the log(OR) of the top hits in MCCS and EPIC-Italy.

**Supplementary Figure 2** Trend of M methylation levels by smoking categories in selected CpGs in the EPIC-Italy study.

**Supplementary Figure 3** Trend of M methylation levels by smoking categories in selected CpGs in the MCCS study.

**Supplementary Figure 4** ROC curves and AUCs corresponding to logistic models with and without the 6 CpGs identified in addition to reported smoking history separately for MCCS and EPIC-Italy.
