# Supplementary material for: DNA methylation changes measured in pre‐diagnostic peripheral blood samples are associated with smoking and lung cancer risk
Source: Int J Cancer. 2016 Oct 11;140(1):50–61. doi: 10.1002/ijc.30431 (PMC5731426; doi:10.1002/ijc.30431)
Supplement: Supplementary file 2 — Supporting Figure Legend [file IJC-140-50-s002.docx]

**LEGEND To Supplementary Figures**

**Supplementary Figure 1** Results of the MWAS in MCCS and EPIC-Italy. (Left panel) Manhattan plot of the log(OR) of the association between lung cancer risk and a 1 SD increment in the methylation level of each CpG. (Right panel) Comparison between the log(OR) of the top hits in MCCS and EPIC-Italy.

**Supplementary Figure 2** Trend of M methylation levels by smoking categories in selected CpGs in the EPIC-Italy study.

**Supplementary Figure 3** Trend of M methylation levels by smoking categories in selected CpGs in the MCCS study.

**Supplementary Figure 4** ROC curves and AUCs corresponding to logistic models with and without the 6 CpGs identified in addition to reported smoking history separately for MCCS and EPIC-Italy.
