# Supplementary figures and images for: DNA methylation changes measured in pre‐diagnostic peripheral blood samples are associated with smoking and lung cancer risk
Source: Int J Cancer. 2016 Oct 11;140(1):50–61. doi: 10.1002/ijc.30431 (PMC5731426; doi:10.1002/ijc.30431)

**Manhattan Plot**

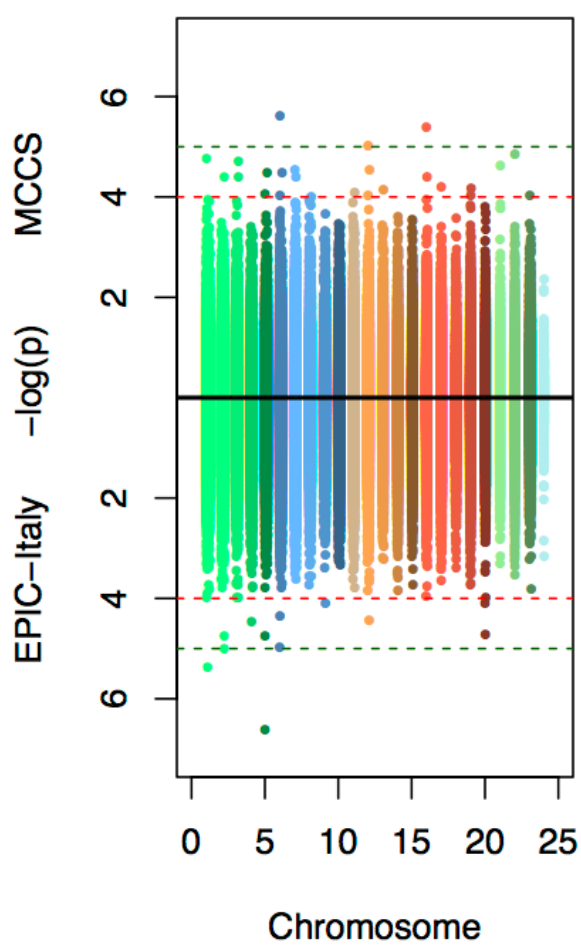

**Top CpGs**

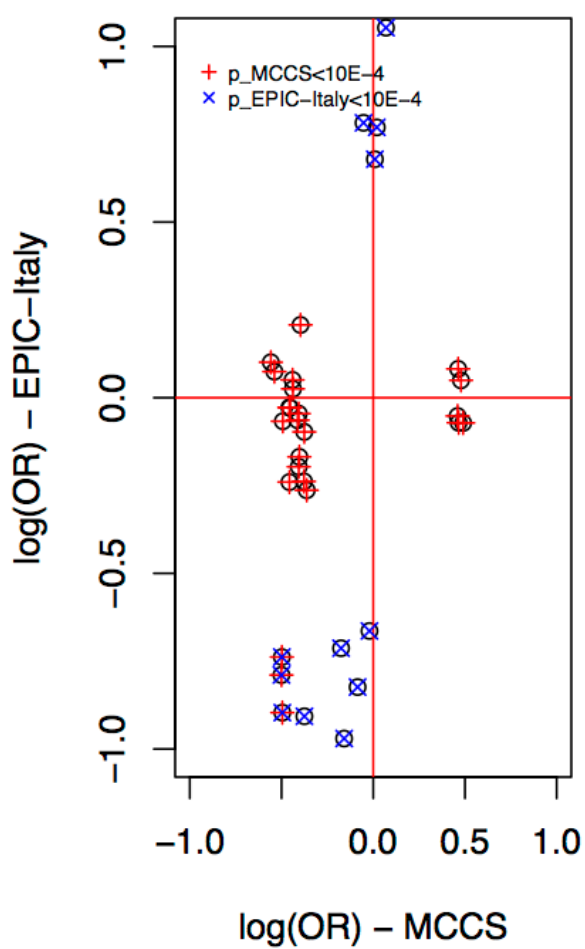

Supplement: Supplementary file 3 — Supporting Figure 1 [file IJC-140-50-s003.pdf]

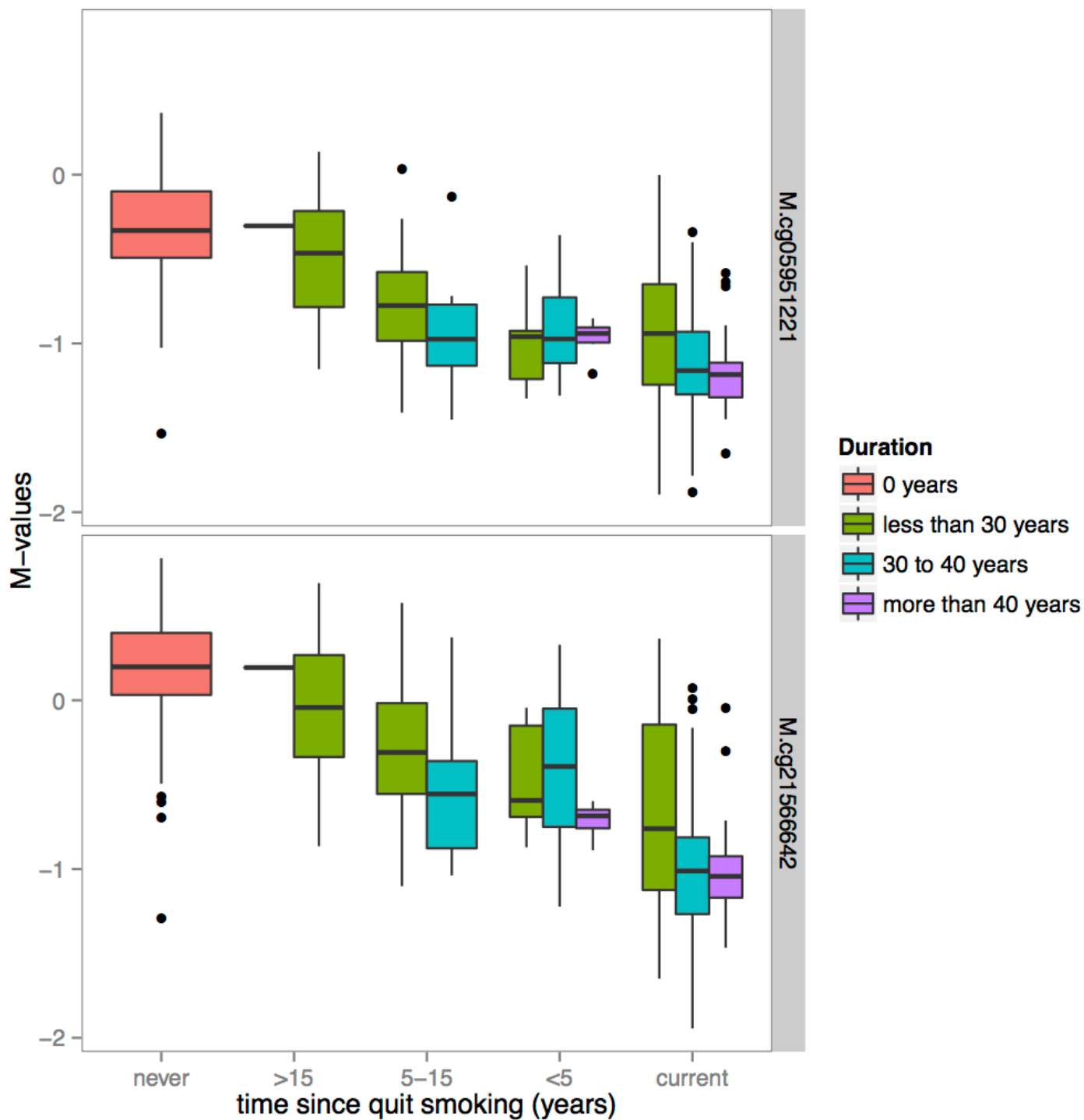

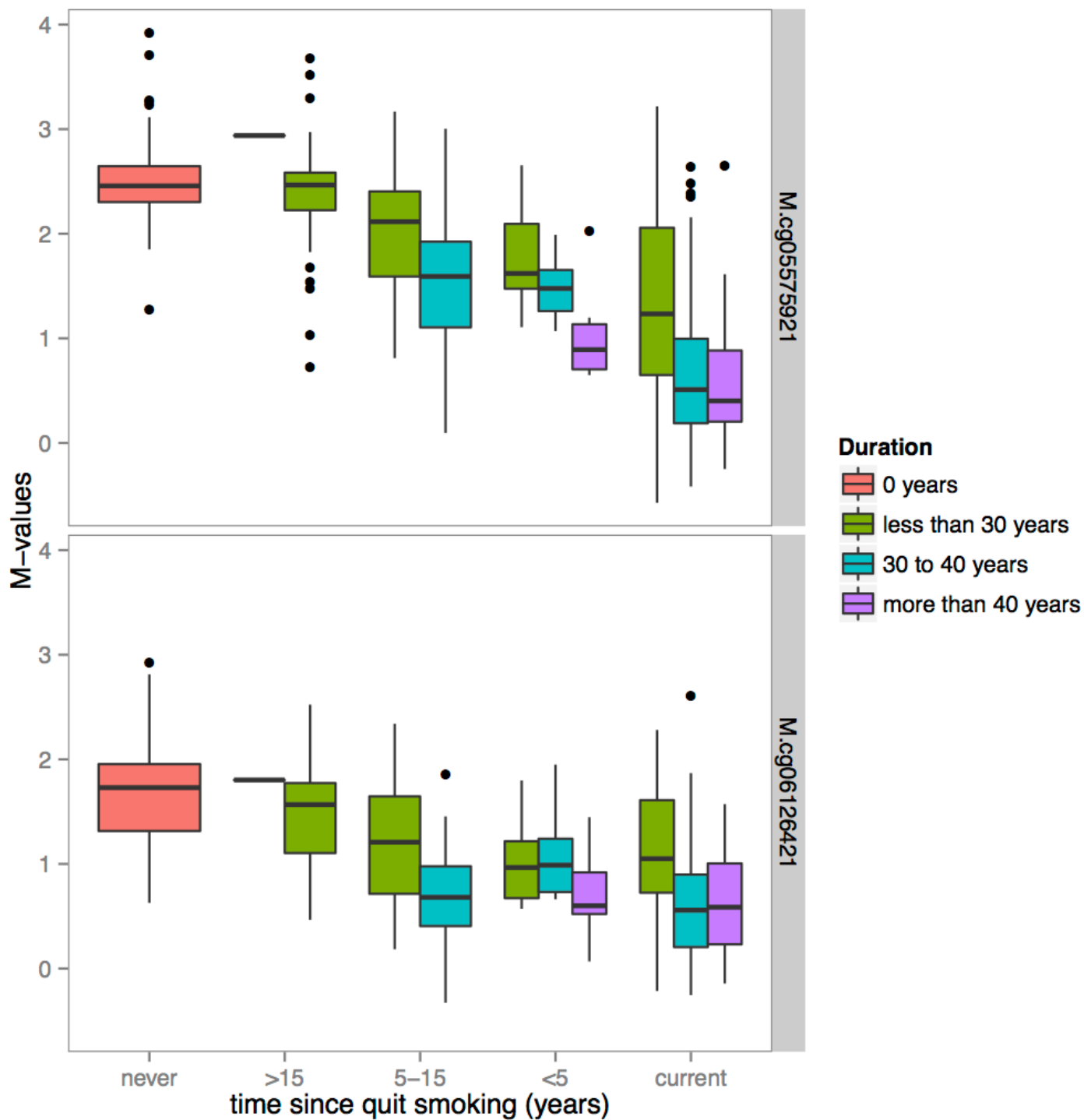

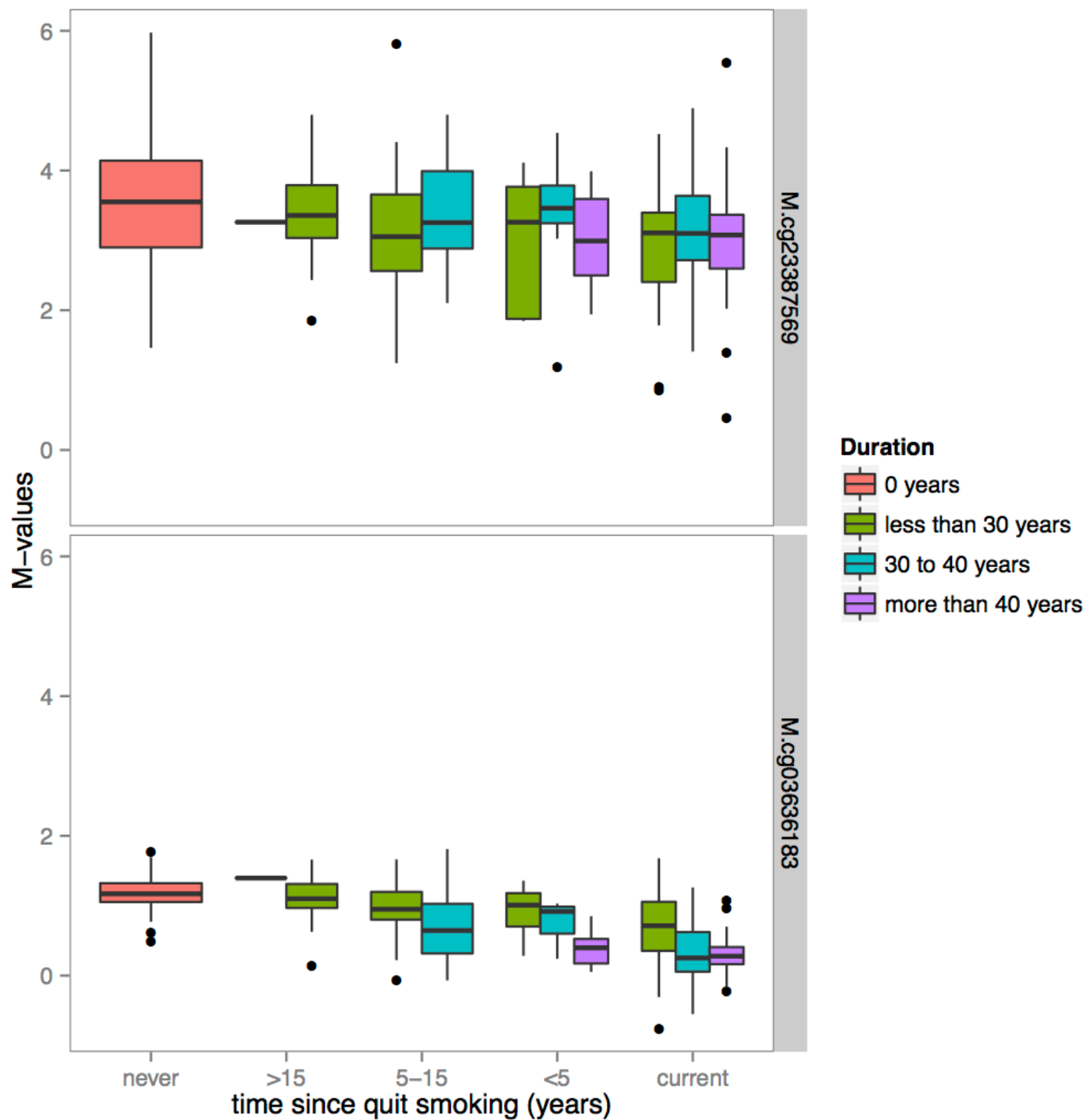

Supplement: Supplementary file 4 — Supporting Figure 2 [file IJC-140-50-s004.pdf]

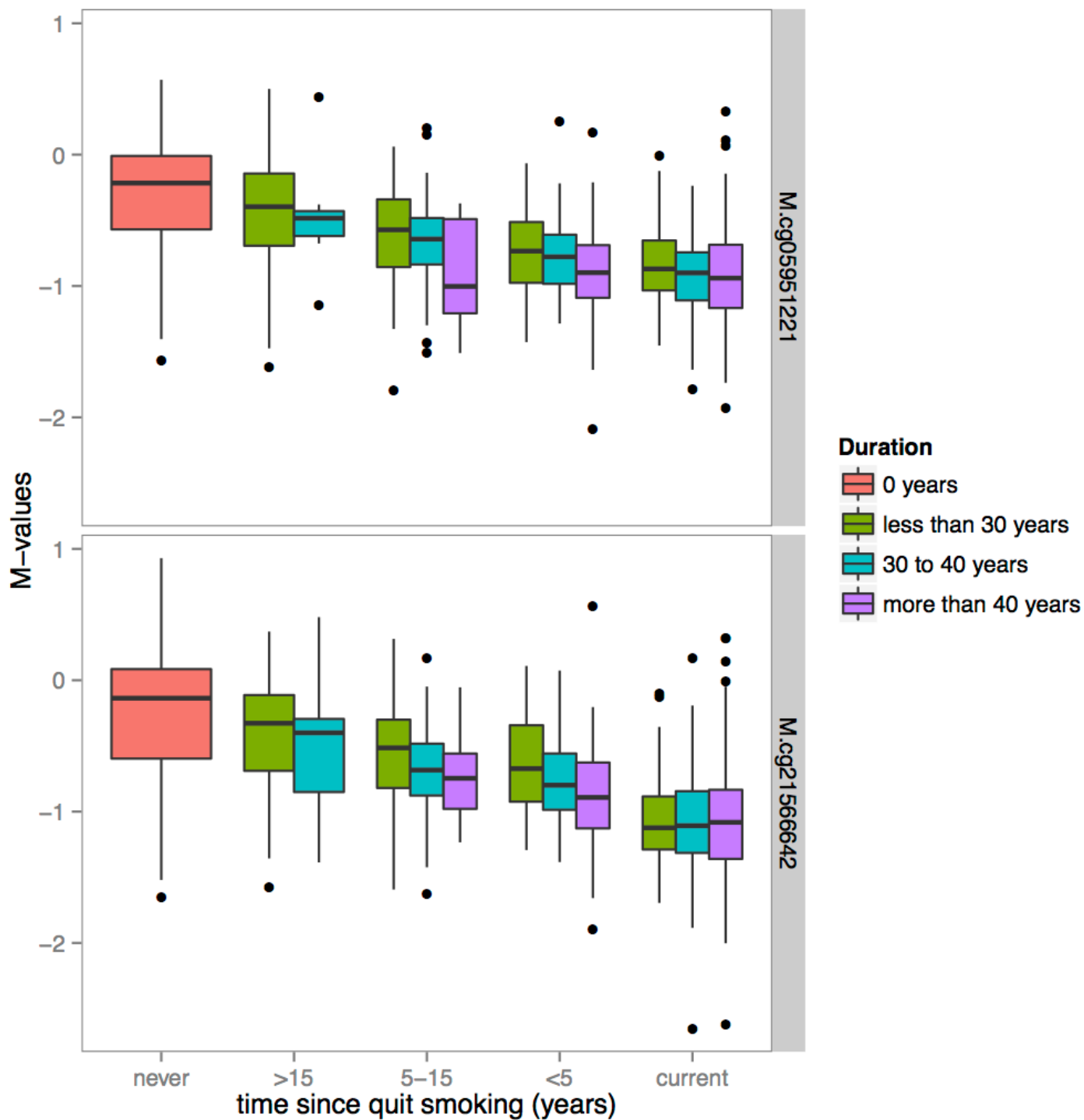

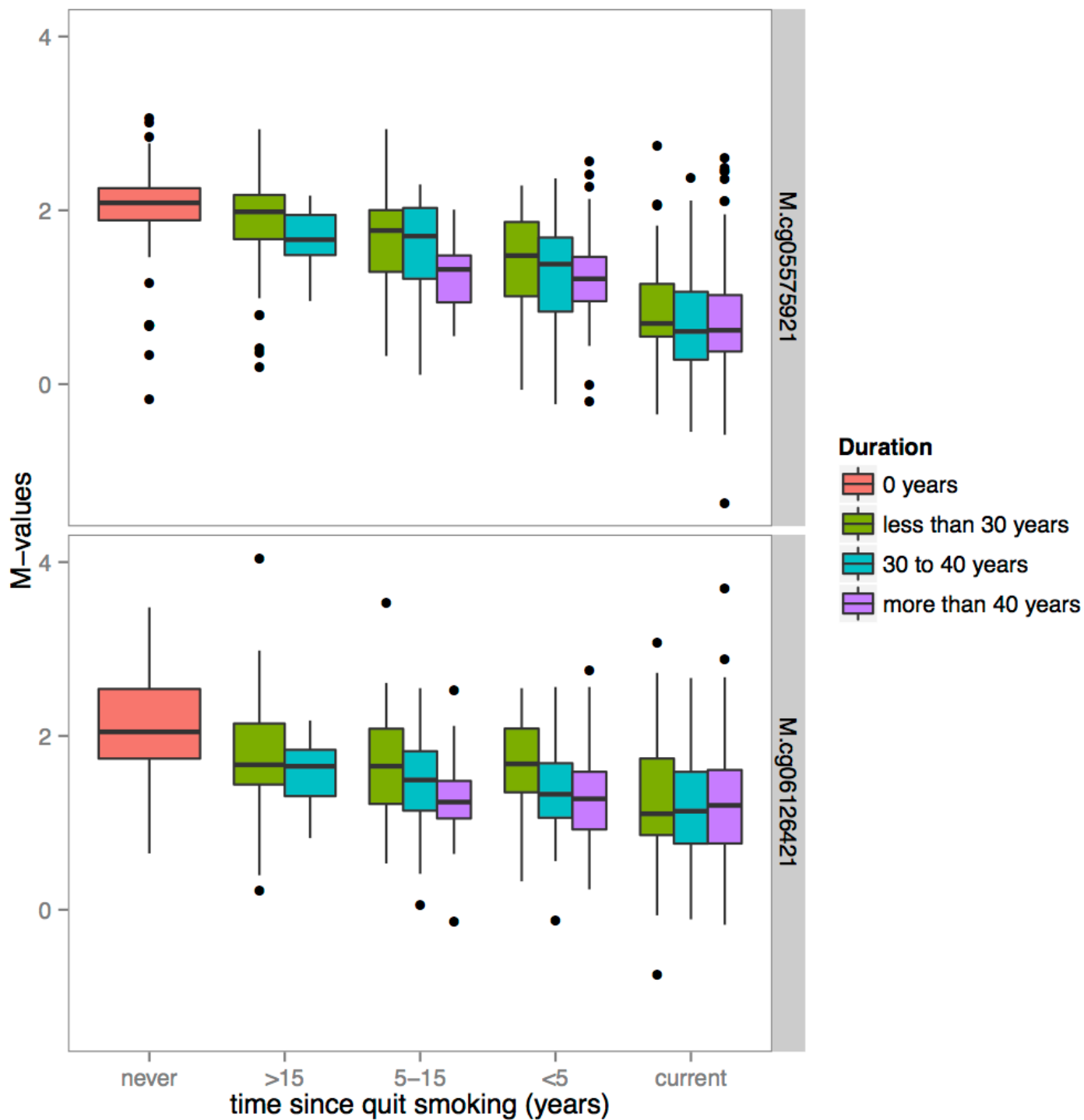

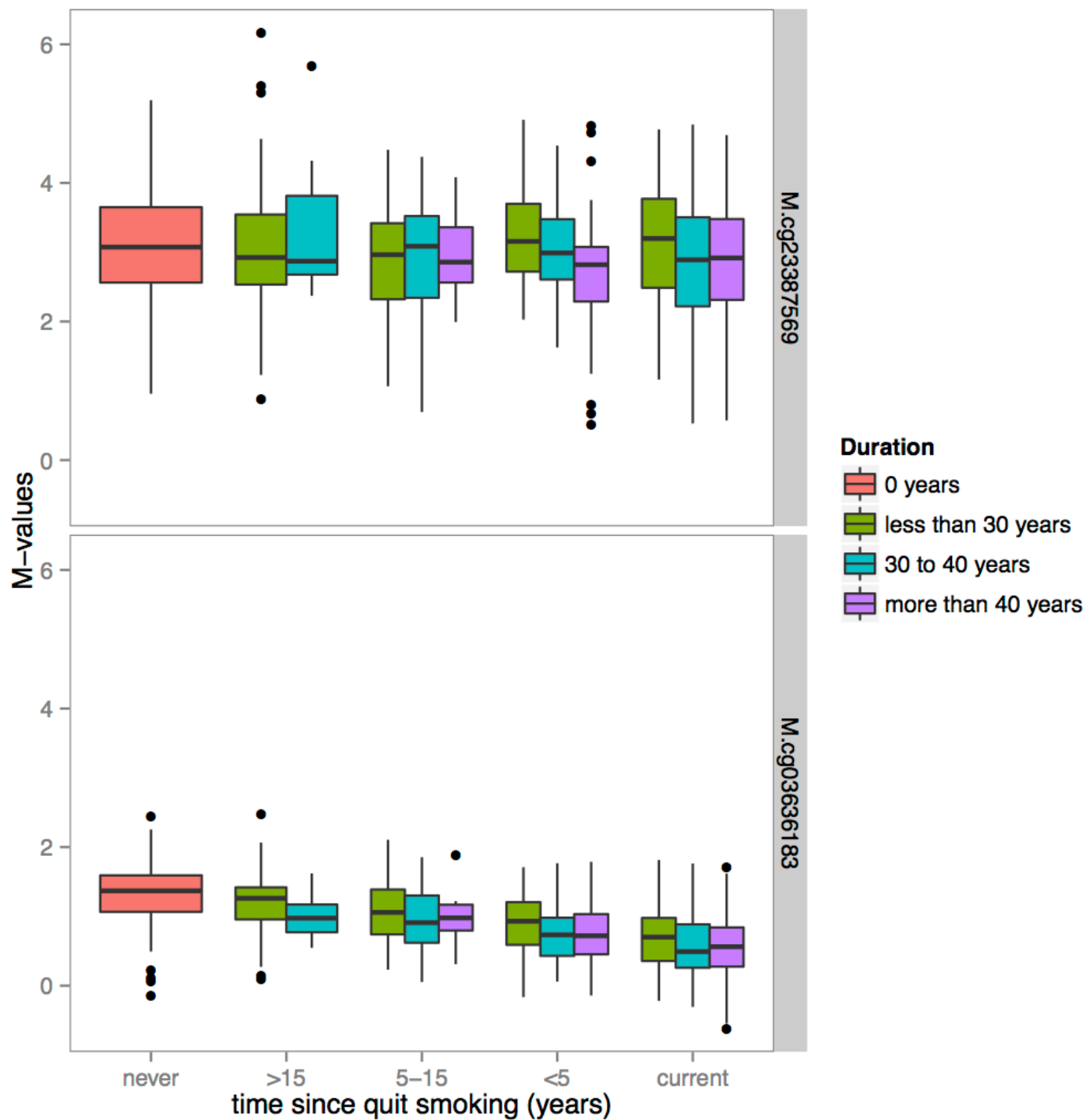

Supplement: Supplementary file 5 — Supporting Figure 3 [file IJC-140-50-s005.pdf]
