# Supplementary material for: DNA methylation changes measured in pre‐diagnostic peripheral blood samples are associated with smoking and lung cancer risk
Source: Int J Cancer. 2016 Oct 11;140(1):50–61. doi: 10.1002/ijc.30431 (PMC5731426; doi:10.1002/ijc.30431)
Supplement: Supplementary file 6 — Supporting Figure 4 [file IJC-140-50-s006.pdf]

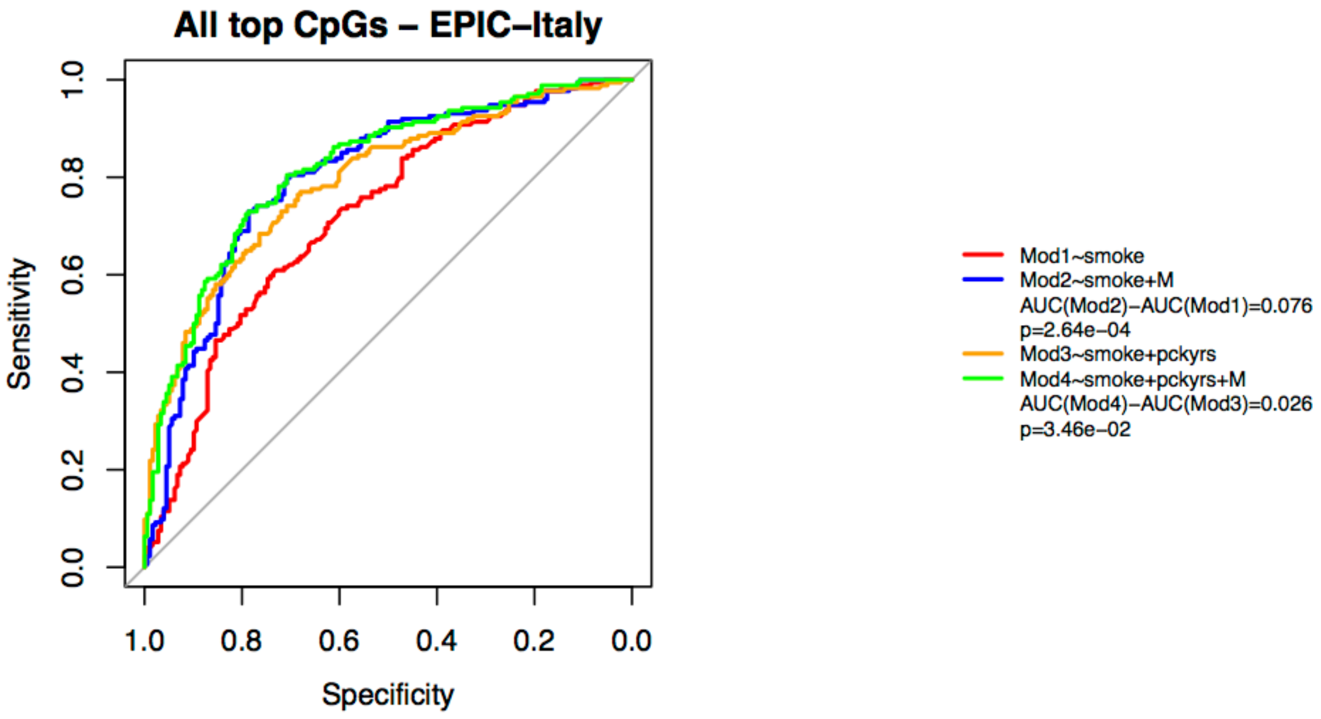
All top CpGs – EPIC–Italy
Sensitivity
Specificity
1.0
0.8
0.6
0.4
0.2
0.0
Mod1~smoke
Mod2~smoke+M
AUC(Mod2)–AUC(Mod1)=0.076
p=2.64e–04
Mod3~smoke+pckyrs
Mod4~smoke+pckyrs+M
AUC(Mod4)–AUC(Mod3)=0.026
p=3.46e–02

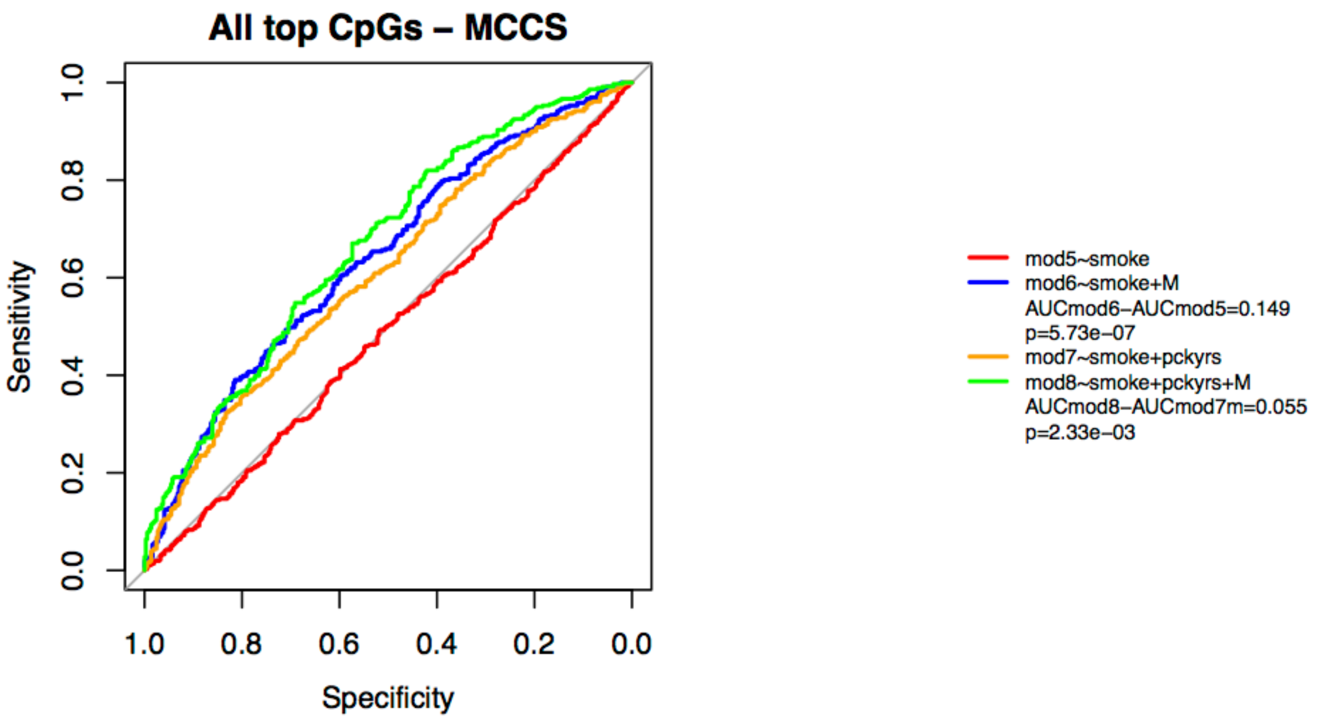
All top CpGs – MCCS
Sensitivity
Specificity
1.0
0.8
0.6
0.4
0.2
0.0
mod5~smoke
mod6~smoke+M
AUCmod6–AUCmod5=0.149
p=5.73e–07
mod7~smoke+pckyrs
mod8~smoke+pckyrs+M
AUCmod8–AUCmod7m=0.055
p=2.33e–03
