# Supplementary material for: DNA methylation changes measured in pre‐diagnostic peripheral blood samples are associated with smoking and lung cancer risk
Source: Int J Cancer. 2016 Oct 11;140(1):50–61. doi: 10.1002/ijc.30431 (PMC5731426; doi:10.1002/ijc.30431)
Supplement: Supplementary file 7 — Supporting Table 1 [file IJC-140-50-s007.pdf]

Supplementary Table 1: OR of lung cancer for 1SD methylation level in each CpG without and with adjustment for cell count according to the method proposed by Houseman in the 5 studies.
For MCCS are presented results for all samples and results for Guthrie Cards (GC).

CpG		MCCS			MCCS-GC			EPIC			NOWAC			NSHDS			Heidelberg		
		OR (95% CI)	p	p lrtest	OR (95% CI)	p	p lrtest	OR (95% CI)	p	p lrtest	OR (95% CI)	p	p lrtest	OR (95% CI)	p	p lrtest	OR (95% CI)	p	p lrtest
cg05951221	not adjusted for cell count	0.69 (0.55-0.86)	9.01E-04		0.65 (0.49-0.86)	2.17E-03		0.4 (0.27-0.61)	1.89E-05		0.6 (0.35-1.02)	5.76E-02		0.54 (0.4-0.72)	4.97E-05		0.64 (0.35-1.18	1.54E-01	
	adjusted for cell count	0.69 (0.55-0.87)	2.02E-03	5.05E-01	0.64 (0.48-0.85)	2.36E-03	9.78E-01	0.39 (0.26-0.6)	2.05E-05	5.45E-01	0.71 (0.38-1.33)	2.89E-01	2.31E-01	0.55 (0.41-0.75)	1.60E-04	3.63E-01	0.63 (0.32-1.	1.60E-01	0.327
cg21566642	not adjusted for cell count	0.61 (0.48-0.77)	3.88E-05		0.62 (0.47-0.83)	1.14E-03		0.45 (0.32-0.64)	9.97E-06		0.54 (0.32-0.9)	1.73E-02		0.46 (0.34-0.64)	3.29E-06		0.54 (0.3-0.98	3.94E-02	
	adjusted for cell count	0.6 (0.47-0.78)	7.24E-05	4.75E-01	0.61 (0.46-0.83)	1.40E-03	9.90E-01	0.46 (0.32-0.65)	1.93E-05	7.68E-01	0.63 (0.37-1.1)	1.08E-01	2.98E-01	0.45 (0.32-0.63)	3.57E-06	1.69E-01	0.49 (0.27-0.	2.27E-02	0.213
cg05575921	not adjusted for cell count	0.61 (0.48-0.78)	8.49E-05		0.67 (0.51-0.89)	5.33E-03		0.41 (0.29-0.57)	2.48E-07		0.37 (0.19-0.71)	2.87E-03		0.43 (0.31-0.6)	4.91E-07		0.72 (0.36-1.46	3.69E-01	
	adjusted for cell count	0.6 (0.46-0.78)	1.52E-04	4.70E-01	0.67 (0.5-0.9)	7.53E-03	9.96E-01	0.39 (0.27-0.56)	4.36E-07	7.44E-01	0.42 (0.2-0.85)	1.57E-02	3.79E-01	0.43 (0.31-0.61)	1.16E-06	3.60E-01	0.53 (0.24-1.	1.27E-01	0.173
cg06126421	not adjusted for cell count	0.61 (0.49-0.75)	2.32E-06		0.64 (0.5-0.82)	3.80E-04		0.48 (0.34-0.68)	4.49E-05		0.58 (0.37-0.91)	1.86E-02		0.65 (0.51-0.82)	3.51E-04		0.22 (0.1-0.5)	3.54E-04	
	adjusted for cell count	0.58 (0.46-0.73)	4.52E-06	4.90E-01	0.62 (0.47-0.8)	3.54E-04	9.63E-01	0.42 (0.29-0.63)	2.31E-05	3.17E-01	0.76 (0.44-1.34)	3.47E-01	4.76E-01	0.6 (0.46-0.79)	2.77E-04	1.30E-01	0.23 (0.1-0.5	6.60E-04	0.606
cg23387569	not adjusted for cell count	0.67 (0.56-0.8)	9.51E-06		0.66 (0.54-0.82)	1.82E-04		0.82 (0.6-1.12)	2.20E-01		0.76 (0.49-1.18)	2.20E-01		0.84 (0.68-1.03)	9.17E-02		0.54 (0.3-0.98	3.94E-02	
	adjusted for cell count	0.67 (0.56-0.81)	2.08E-05	5.10E-01	0.67 (0.53-0.83)	3.23E-04	1.00E+00	0.85 (0.61-1.18)	3.31E-01	5.88E-01	0.95 (0.58-1.56)	8.36E-01	2.50E-01	0.86 (0.7-1.07)	1.68E-01	2.19E-01	0.40 (0.27-0.	2.27E-02	0.213
cg12312863	not adjusted for cell count	0.63 (0.52-0.77)	4.00E-06		0.59 (0.46-0.75)	1.82E-05		0.79 (0.58-1.07)	1.21E-01		0.92 (0.64-1.33)	6.70E-01		1.17 (0.92-1.48)	2.04E-01		0.64 (0.35-1.	1.50E-01	
	adjusted for cell count	0.61 (0.5-0.74)	1.29E-06	1.44E-01	0.58 (0.46-0.75)	1.70E-05	9.58E-01	0.78 (0.57-1.07)	1.24E-01	5.17E-01	0.96 (0.64-1.42)	8.32E-01	1.00E-01	1.15 (0.9-1.46)	2.65E-01	1.81E-01	0.63 (0.32-1.	1.60E-01	0.327
cg03636183	not adjusted for cell count	0.67 (0.54-0.84)	3.63E-04		0.78 (0.62-0.98)	3.42E-02		0.59 (0.45-0.78)	1.97E-04		0.47 (0.29-0.78)	3.22E-03		0.52 (0.39-0.69)	9.70E-06		0.66 (0.68-1.16	1.46E-01	
	adjusted for cell count	0.67 (0.53-0.84)	5.90E-04	4.35E-01	0.78 (0.61-1)	4.83E-02	9.94E-01	0.6 (0.45-0.8)	5.38E-04	8.10E-01	0.52 (0.3-0.91)	2.13E-02	4.12E-01	0.5 (0.37-0.69)	1.80E-05	2.41E-01	0.62 (0.35-1.	1.12E-01	0.273
