# Supplementary material for: DNA methylation changes measured in pre‐diagnostic peripheral blood samples are associated with smoking and lung cancer risk
Source: Int J Cancer. 2016 Oct 11;140(1):50–61. doi: 10.1002/ijc.30431 (PMC5731426; doi:10.1002/ijc.30431)
Supplement: Supplementary file 8 — Supporting Table 2 [file IJC-140-50-s008.pdf]

Supplementary Table 2: distribution of cell count in MCCS and EPICcalculated with the method proposed by Houseman by smoking status and in MCCS by type of biospecimen

**MCCS**		N	CD8T	%delta	CD4T	%delta	NK	%delta	Bcell	%delta	Mono	%delta	Gran	%delta
all		734	0.07		0.21		0.10		0.09		0.07		0.51	
BC/Lymph		230	0.09	0.43	0.23	0.14	0.15	0.70	0.10	0.22	0.08	0.14	0.41	0.27
GC		504	0.06		0.20		0.08		0.08		0.07		0.55	
never		86	0.09	0.43	0.22	0.10	0.12	0.20	0.10	0.22	0.07	0.14	0.46	0.12
former		314	0.06		0.20		0.11		0.08		0.08		0.52	
current		334	0.07		0.22		0.10		0.09		0.08		0.50	
never	BC/Lymph	38	0.11	0.33	0.25	0.13	0.15	0.13	0.12	0.30	0.06	0.38	0.37	0.15
former	BC/Lymph	92	0.08		0.22		0.16		0.09		0.07		0.43	
current	BC/Lymph	100	0.09		0.24		0.14		0.11		0.09		0.40	
never	GC	48	0.07	0.17	0.20	0.10	0.09	0.13	0.09	0.13	0.07	0.14	0.53	0.05
former	GC	222	0.06		0.19		0.09		0.08		0.08		0.56	
current	GC	234	0.06		0.21		0.08		0.09		0.07		0.55	

**EPIC**			CD8T	%delta	CD4T	%delta	NK	%delta	Bcell	%delta	Mono	%delta	Gran	%delta
all		376	0.07		0.21		0.10		0.09		0.07		0.51	
never		108	0.07	0.00	0.22	0.05	0.11	0.10	0.09	0.00	0.07	0.14	0.50	0.02
former		124	0.07		0.22		0.10		0.09		0.07		0.51	
current		141	0.07		0.21		0.10		0.09		0.08		0.51	
