# Supplementary material for: DNA methylation changes measured in pre‐diagnostic peripheral blood samples are associated with smoking and lung cancer risk
Source: Int J Cancer. 2016 Oct 11;140(1):50–61. doi: 10.1002/ijc.30431 (PMC5731426; doi:10.1002/ijc.30431)
Supplement: Supplementary file 9 — Supporting Table 3 [file IJC-140-50-s009.pdf]

Supplementary Table 3: OR of lung cancer for 1 SD methylation level in each CpG by time since blood collection overall and by smoking status. Results in the MCCS study.

For the analisis on all subjects:		<5 years		[5-10) years		[10-15) years		15 yearsor more		
For the analysis by smoking status:		<10 years		10 years or more						
TargetID		OR (95% CI)	p-value	OR (95% CI)	p-value	OR (95% CI)	p-value	OR (95% CI)	p-value	p.het
cg05951221	All	0.58 (0.36-0.94)	2.81E-02	0.74 (0.51-1.08)	1.15E-01	0.68 (0.46-1)	4.76E-02	0.77 (0.44-1.34)	3.52E-01	0.86
	Never smokers	0.63 (0.16-2.51)	5.12E-01	0.51 (0.19-1.33)	1.67E-01					0.81
	Former smokers	0.6 (0.35-1.04)	6.88E-02	0.58 (0.31-1.07)	8.03E-02					0.93
	Current smokers	0.86 (0.58-1.3)	4.79E-01	0.88 (0.52-1.49)	6.37E-01					0.95
cg21566642	All	0.55 (0.35-0.88)	1.19E-02	0.7 (0.47-1.05)	8.57E-02	0.61 (0.41-0.9)	1.34E-02	0.47 (0.23-0.95)	3.44E-02	0.73
	Never smokers	1.35 (0.46-4)	5.88E-01	0.49 (0.19-1.32)	1.60E-01					0.17
	Former smokers	0.56 (0.32-0.96)	3.59E-02	0.47 (0.23-0.97)	4.02E-02					0.71
	Current smokers	0.82 (0.5-1.32)	4.08E-01	0.62 (0.35-1.08)	9.21E-02					0.45
cg05575921	All	0.57 (0.35-0.93)	2.51E-02	0.6 (0.39-0.91)	1.68E-02	0.68 (0.47-0.99)	4.60E-02	0.45 (0.2-1)	4.97E-02	0.76
	Never smokers	1 (0.08-12.18)	1.00E+00	0.46 (0.13-1.62)	2.25E-01					0.58
	Former smokers	0.46 (0.25-0.82)	9.06E-03	0.74 (0.39-1.41)	3.57E-01					0.26
	Current smokers	0.72 (0.47-1.12)	1.50E-01	0.74 (0.43-1.25)	2.62E-01					0.95
cg06126421	All	0.42 (0.25-0.69)	6.54E-04	0.67 (0.46-0.98)	3.83E-02	0.61 (0.43-0.86)	5.02E-03	0.78 (0.49-1.24)	2.92E-01	0.29
	Never smokers	0.69 (0.21-2.21)	5.28E-01	0.36 (0.11-1.24)	1.06E-01					0.47
	Former smokers	0.6 (0.37-0.98)	4.02E-02	0.71 (0.42-1.2)	2.02E-01					0.64
	Current smokers	0.64 (0.41-0.99)	4.42E-02	0.7 (0.48-1.03)	7.40E-02					0.75
cg23387569	All	0.59 (0.39-0.88)	1.02E-02	0.81 (0.6-1.11)	1.88E-01	0.54 (0.39-0.76)	2.95E-04	0.79 (0.5-1.25)	3.14E-01	0.25
	Never smokers	0.67 (0.28-1.61)	3.76E-01	0.45 (0.2-1.01)	5.38E-02					0.50
	Former smokers	0.42 (0.24-0.74)	2.74E-03	0.53 (0.31-0.9)	1.84E-02					0.56
	Current smokers	0.86 (0.63-1.17)	3.46E-01	0.7 (0.47-1.03)	7.09E-02					0.41
cg12312863	All	0.54 (0.36-0.83)	4.35E-03	0.62 (0.44-0.89)	9.50E-03	0.66 (0.49-0.9)	7.98E-03	0.76 (0.44-1.31)	3.24E-01	0.79
	Never smokers	0.55 (0.2-1.49)	2.38E-01	0.64 (0.28-1.44)	2.82E-01					0.81
	Former smokers	0.57 (0.33-0.97)	3.77E-02	0.69 (0.44-1.1)	1.22E-01					0.57
	Current smokers	0.53 (0.36-0.78)	1.15E-03	0.57 (0.36-0.9)	1.52E-02					0.78
cg03636183	All	0.73 (0.47-1.12)	1.47E-01	0.66 (0.45-0.97)	3.58E-02	0.72 (0.51-1.02)	6.35E-02	0.49 (0.25-0.92)	2.77E-02	0.71
	Never smokers	0.9 (0.31-2.57)	8.42E-01	0.64 (0.27-1.5)	3.03E-01					0.62
	Former smokers	0.56 (0.33-0.97)	3.89E-02	0.78 (0.46-1.31)	3.46E-01					0.38
	Current smokers	0.94 (0.61-1.45)	7.76E-01	0.66 (0.4-1.09)	1.02E-01					0.27
